# CC48 a new CB2R agonist/FAAH inhibitor dual drug blocks gastric cancer progression and overcomes paclitaxel resistance

**DOI:** 10.1186/s13046-025-03476-7

**Published:** 2025-07-16

**Authors:** Annalisa Schirizzi, Natasha Renna, Giampiero De Leonardis, Rosangela Montanaro, Francesco Mastropasqua, Giovanni Graziano, Chiara Riganti, Isabella Pisano, Antonio Laghezza, Carmen Abate, Angela Stefanachi, Nicola Antonio Colabufo, Cristina Caccioppoli, Giusy Bianco, Anna Maria Valentini, Raffaele Armentano, Gianluigi Giannelli, Marialessandra Contino, Rosalba D’Alessandro

**Affiliations:** 1 National Institute of Gastroenterology, IRCCS “Saverio de Bellis” Research Hospital, Castellana Grotte, BA 70013 Italy; 2 Department of Pharmacy-Pharmaceutical Sciences, University of Bari, Bari, BA 70125 Italy; 3 Department of Oncology, University of Torino, Torino, Italy; 4 Department of Bioscience, Biotechnology and Environment, University of Bari Aldo Moro, Bari, 70125 Italy; 5 Department of Emergency and Organ Transplantation, University of Bari Aldo Moro, Bari, 70124 Italy

**Keywords:** Gastric cancer treatment, Cannabinoid receptor subtype 2 (CB2R), CB2R ligands, Paclitaxel-resistance, Novel target therapy

## Abstract

Gastric cancer (GC) has poor survival in advanced stages, with limited treatment options. Paclitaxel (PTX) is commonly used, but resistance often arises, highlighting the need for targeted therapies. Cannabinoid receptor type 2 (CB2R) is overexpressed in several cancers and its activation has been associated with reduced tumor growth and metastasis. This study evaluated the antitumor activity of selected CB2R agonists with dual activity (**CC48** and **Fi9**) compared to single-target compounds (**ASF151**), a reference agonist (**compound 1**), and an antagonist (**AM630**). The compounds’ cytotoxicity was determined in GC lines, including PTX-resistant cells, with different levels of CB2R expression. Firstly, were ported that the addition of CB2R ligands to PTX significantly reduces the actively proliferating cells (Ki67+) even in chemotherapy-resistant GC cells. Concentrations below the IC50 of all compounds were used to minimise toxicity. Activation of Akt/mTORC1 and MAPK cascades were found to be related to antiproliferative activity, which was found to be independent of CB2R expression in the different cell lines. Surprisingly, both agonist and antagonist compounds inhibited cell growth. The interaction of **CC48** and the reference compounds **1** and **AM630**, with P-glycoprotein (P-gp) could explain their greater effectiveness in overcoming PTX resistance. Furthermore, **CC48** was particularly effective among the agonists in inducing the expression of key autophagy proteins and activating the apoptotic pathway via caspase 3/7 (*p* < 0.05). The combination of **CC48** with PTX further amplified this effect in both sensitive and resistant cells (*p* < 0.01). **CC48** significantly reduced GC cells migration and epithelial-mesenchymal transition (EMT) by modulating the vimentin protein (*p* < 0.05). In an orthotopic mouse model, **CC48** inhibits tumor volume (*p* < 0.01)and also reduces the number of Ki67 + cells (*p* < 0.05), without cytotoxic effects. Histological analysis revealed widespread necrosis with inflammatory and apoptotic features, including pyknotic nuclei and fibrotic replacement in **CC48-**treatedtumors. Moreover, **CC48** treatment reduced circulating levels of G-CSF, IL-12 (p40), and eotaxin (*p* < 0.05), suggesting an immunomodulatory role. In conclusion **CC48**, a novel multi-target ligand (MTDL), activating CB2R and inhibiting Fatty Acid Amide Hydrolase (FAAH), effectively blocks GC progression modulating the immune response and overcoming PTX resistance.

**Supplementary Information:**

The online version contains supplementary material available at 10.1186/s13046-025-03476-7.

## Background

Gastric Cancer (GC) accounts for about 1.5% of all new cancers diagnosed in the US each year [cancer.org]. In Western countries, 80% of patients are diagnosed with unresectable advanced disease or develop a recurrence within 5 years after surgery with curative intent. Hence worldwide, the prognosis of advanced disease remains poor, with a 5-year survival rate of < 30%, and < 4% for metastatic disease [1, 2]. Due to the aggressive nature of the disease and the problem of late stage at diagnosis, the search for predictive/prognostic markers has a crucial role in the treatment of locally, advanced and metastatic GC. Currently, despite advances in immunotherapy and new molecular targeted therapies, treatment options available for these patients are limited. Chemotherapy remains the cornerstone of treatment for advanced GC, with a median survival of about one year [3].Paclitaxel (PTX) is used as front-line chemotherapy in advanced disease, in combination with two or more chemotherapy agents and in combination with Ramucirumab in second line therapy [4, 5]. The association of PTX and Ramucirumab seems to be a promising option to prevent resistance to PTX also in recurrent and metastatic GC patients receiving taxane-based first-line palliative chemotherapy [6]. PTX is a taxane-derived antimitotic drug with microtubules stabilization mechanism of action. Microtubules disorganization decreases responses to antimitotic treatments and apoptotic process failure can induce acquired resistance to PTX in advanced GC [7, 8]. Notably, a new biomarker for tumor growth and metastasis reported in different types of solid tumors is Cannabinoid Receptor subtype 2 (CB2R), a G protein-coupled receptor**(**GPCR)belonging to the endocannabinoid system. CB2R overexpression has been widely reported in inflammatory states and in cancer onset, and its activation has been shown to elicit different anti-tumor mechanisms in several cancer types [9–13]. Specifically, in colorectal cancer (CRC) cell models of resistance to oxaliplatin, the standard chemotherapy, Cannabidiol (CBD), a CB2R modulator, reduced drug resistance through the enhancement of autophagy [14] and recently, Iden and collaborators demonstrated that CB2R activation reduced the levels of immunosuppressive factors in the CRC tumor-microenvironment [15].The goal of the present study was to elucidate the CB2R-mediated mechanisms underlying the growth and spread of gastric cancer (GC), and to evaluate the efficacy of a multitarget strategy that promotes both direct (CB2R agonism) and indirect (inhibition of the enzyme responsible for endocannabinoid degradation) activation of CB2R as a potential approach to enhance systemic treatment. For this reason, we selected two CB2R reference compounds displaying opposite activities at the CB2R, the CB2R single target agent **compound 1**, with a known CB2R agonist activity [16], and compound **AM630** with an antagonist activity [17–20].Three CB2R agonists belonging to our library of CB2R compounds were also selected, **CC48**, **Fi9** and **ASF151**, bearing a similar chemical scaffold and a different affinity and selectivity profile towards the two cannabinoid receptor subtypes 1 and 2 (CB1R and CB2R) [21, 22] and a reported multitarget (**CC48** and **Fi9**) and single target (**ASF151**) activity at the CB2R. The two compounds **CC48** and **Fi9** are classified as MultiTarget Directed Ligands (MTDLs) due to their dual activity. Specifically, they act as agonists at the CB2 receptor (CB2R) and inhibit Fatty Acid Amide Hydrolase (FAAH), the enzyme responsible for the degradation of endocannabinoids [21]. These two compounds exhibit high affinity for CB2R and good selectivity, although a certain degree of affinity for the CB1R receptor and potential agonist activity cannot beexcluded. Indeed, **CC48** exhibits a 16-fold higher affinity for CB2R compared to CB1R, while **Fi9** shows a 3-fold higher affinity for CB2R over CB1R. **ASF151** was selected as a single target CB2R agonist with a good affinity and high selectivity for CB2R, due to the absence of affinity/activity at CB1R. All these compounds (**CC48**, **Fi9** and **ASF151**) exhibited anti-inflammatory activity, being able to inhibit the production of pro-inflammatory cytokines (TNF-α, IL-1β, IFN-γ, IL-6) and induce the production of the anti-inflammatory types (IL-10, IL-4) [21, 22]. As it is widely demonstrated that inflammation is linked to the occurrence of pathological changes including cancer, we selected these compounds as potential CB2R anti-tumor agents in GC. An extensive analysis was performed of the cytotoxic action of the selected CB2R ligands, together with their effects on cell growth, by assessing the main pathways involved in proliferation, autophagy and apoptosis. The induction of oxidative stress and the effects of the compounds on cell migration rates were also investigated. To explore the role of CB2R in PTX-mediated resistance, a PTX-resistant cell model was used. Based on in vitro analysis, the most effective CB2R ligand **CC48** was selected and subsequentlytested in orthotopic mouse models generated by intraperitoneal inoculation of a human gastric carcinoma cell line to further evaluate the molecule’s efficacy and potential side effects.

## Methods

### In vitro experiments

#### Cells and drugs

HGC27, AGS, NCl-N87 and KATO III, human gastric cancer cell lines were purchased and authenticated by American Type Culture Collection (Manassas, Virginia, USA). All cell culture components were purchased from Sigma-Aldrich (Milan, Italy) and Celbios.r.l. (Milano, Italy). PTX-resistant HGC27 (HGC27-R) were obtained by exposure to increasing concentrations of the chemotherapy agent, starting with a concentration of 1/60th of the IC_50_ and continuing with subcultures, increasing the concentration by 25% every fortnight [23]. Cells are considered resistant to PTX when they are able to grow exponentially in the presence of a PTX concentration equal to that of the IC_50_ of the sensitive counterpart. HGC27-R were grown under the same experimental conditions as their sensitive counterpart with the addition of PTX at 9 nM. All experiments comparing resistant and sensitive cell lines were performed at a PTX concentration close to the IC_50_ of the sensitive cells. MDCK-MDR1 was a gift of Prof. P. Borst, NKI-AVL Institute, Amsterdam, The Netherlands. PTX was purchased from Teva Italia S.r.l. (Milan, Italy). **AM630** was purchased from TocrisS.r.l. (Milan, Italy), while derivatives **1**, **CC48**, **Fi9** and **ASF151**, already reported, were synthesized in our laboratories according to the published literature [21, 22]. 2’,7’-dichlorofluorescein diacetate, DCF-DA, was purchased from Sigma-Aldrich (Milan, Italy). Calcein-AM, was obtained from Sigma-Aldrich (Milan, Italy).

#### Cell cultures

HGC27-S/R human gastric cancer cell lines were grown in high glucose supplemented DMEM, while AGS, NCl-N87 and KATO III human gastric cancer cell lines were grown in high glucose supplemented RPMI; both media were enriched with 10% fetal bovine serum, 2 mM glutamine, 100 U/mL penicillin, 100 µg/mL streptomycin. All cell lines were grown in a humidified incubator at 37 °C in a 5% CO_2_ atmosphere. MDCK-MDR1 cells were a gift of Prof. P. Borst, NKI-AVL Institute, Amsterdam, The Netherlands. MDCK-MDR1 cells were grown in high glucose supplemented DMEM with 10% fetal bovine serum, 2 mM glutamine, 100 U/mL penicillin, 100 mg/mL streptomycin, in a humidified incubator at 37 °C in a 5% CO_2_ atmosphere.

#### Gene expression analysis

Total RNA was extracted from GC cells using the Qiagen RNeasy Mini Kit (Qiagen, Hilden, Germany) following the manufacturer’s instructions. Samples were retro-transcribed using the iScript Advanced cDNA Synthesis Kit (Bio-Rad Laboratories, California, USA). The cDNA samples were analyzed by Real-Time-PCR for CB1R and CB2R expression. Experiments were carried out in triplicate using the SsoAdvanced Universal SYBR Green Supermix (Bio-Rad Laboratories, California, USA) on a CFX96 Touch Real-Time PCR Detection System (Bio-Rad Laboratories, California, USA) according to the manufacturer’s instructions. mRNA expression was normalized with the GAPDH housekeeping gene. Pre − validated PrimePCR templates for SYBR Green Assay (Bio-Rad Laboratories, California, USA) were used for reactions. Relative quantification was performed using the ddCT method.

#### FAAH inhibition assay

Assays were performed using 96-well black flat-bottom microtiter NBS plates (COSTAR flat black). The experiments were conducted in a total volume of 200 µl, first incubating different concentrations of each potential inhibitor in an appropriate fluorometric assay buffer (tris-HCl 125 mM, Na_2_EDTA 2H_2_O 1mM, pH = 9.0) with the enzyme (FAAH Human recombinant, Cayman Chemical, Ann Arbor, MI, USA) for 10 min at room temperature, keeping the plate under orbital shaking. The substrate (7-amino-4-methyl-2* H*-1-benzopyran-2-one-5*Z*,8*Z*,11*Z*,14*Z*-eicosatetraen-amide, AMC-AA, 1µM final concentration) was then added, and the assay was incubated for 2 h at 37 °C in a TECAN infinite M1000Pro plate reader (Tecan, Männedorf, Switzerland) which measured the fluorescence from each well every 30 s (λ_ex_ = 340 nm, λ_em_ = 450 nm), determining FAAH activity as relative fluorescence units (RFU). Control wells lacking the inhibitor and blank wells lacking both inhibitor and enzyme were used to calculate the percent inhibition for each tested compound. IC_50_ values were calculated via GraphPad Prism 5.0 (GraphPad Software, La Jolla, CA, USA) and are reported as mean ± SEM of at least three independent measurements performed in triplicate.

#### Cytotoxicity assay

Cell viability was checked with the MTT assay at 48 h and 72 h [24]in HGC27-S/R and AGS gastric cancer cell lines. On day 1, 5000 cells/well were seeded into 96-well plates in a volume of 100 µL. On day 2, each drug was added at the concentrations: 0.1µM, 1µM, 10µM, 30µM, 50µM, 100µM. In all the experiments, the various drug-solvents (ethanol, DMSO) were added in each control to evaluate a possible solvent cytotoxicity. After the established incubation time with drugs (48 h, 72 h), MTT (0.5 mg/mL) was added to each well, and after 3 h incubation at 37 °C, the supernatant was removed. The formazan crystals were solubilized using 100 µL of DMSO and the absorbance values at 570 and 630 nm were determined on the microplate reader Victor 3 from PerkinElmer Life Sciences. Results are expressed as mean ± SD of 3 independent experiments in triplicate.

#### Protein expression analysis

Western Blotting analysis was performed as previously described [25] in both HGC27-S/R and AGS cells. Briefly, for each experimental condition the cells were lysed in ice-cold lysis buffer (50 mM Tris, 10 mM EDTA, 1% v/v Triton-X100), supplemented with the protease/phosphatase inhibitor cocktail set (Merck KGaA, Darmstadt, Germany), incubated on ice for 15 min and centrifuged at 13,000 × g for 15 min at 4 °C. Protein extracts were quantified by Micro BCA™ Protein Assay Kit (Thermo Fisher Scientific Inc., MA USA) and 40 µg of total protein extract was loaded on SDS-PAGE and immunoblotted with the following antibodies: CB1R and CB2R (1:500, rabbit polyclonal, Abcam, Cambridge, UK), P-TSC2 (Ser939) and TSC2 (1:1000Cell Signaling, Beverly, MA, USA), PI3K (1:1000Cell Signaling, Beverly, MA, USA), P-P70 (Thr389) and P70 (1:1000Cell Signaling, Beverly, MA, USA), P-Akt (Ser473 Thr308) and Akt (1:1000Cell Signaling, Beverly, MA, USA), P-S6 (Ser235/236) and S6 (1:1000Cell Signaling, Beverly, MA, USA), 4EBP1(1:1000Cell Signaling, Beverly, MA, USA), P-ERK(Thr202/Tyr204) and ERK (1:1000Cell Signaling, Beverly, MA, USA), PPRγ (1:1000Cell Signaling, Beverly, MA, USA), Phospho-SAPK/JNK (Thr183/Tyr185) and JNK2 (1:1000 Cell Signaling, Beverly, MA, USA), Phospho-c-Jun (Ser63) and c-Jun (1:1000 Cell Signaling, Beverly, MA, USA), caspase 3/7 (1:1000 Cell Signaling, Beverly, MA, USA), Phospho-βcatenin (Ser675) and βcatenin (1:1000 Cell Signaling, Beverly, MA, USA), vimentin (1:1000Cell Signaling, Beverly, MA, USA), P-cofillin (Ser3) and cofillin (1:1000Cell Signaling, Beverly, MA, USA), actin (1:4000Cell Signaling, Beverly, MA, USA). Subsequently, the membranes were incubated with the corresponding horseradish peroxidase (HRP)-conjugated secondary antibodies (Bio-Rad, Hercules, CA, USA). An enhanced chemiluminescence kit (Bio-Rad, Hercules, CA, USA) was used. A Chemidoc XRS + and the Bio-rad software (Bio-Rad, Hercules, CA, USA) was used to observe and analyze the chemiluminescence signals from proteins. Total protein expression was quantified using the ImageJ software (http://rsb.info.nih.gov/ij/). The expression levels of each of the investigated proteins were normalized to the actin level.

#### Ki67 cell proliferation assay

HGC27-S/R cells were treated with 4 nM PTX and 6 µM **AM630**, 10 µM **CC48**, 10 µM **Fi9**, 10 µM **ASF151** and 10 µM **compound 1** administered alone or in combination for 48 h. After the specified drug treatments, the cells were processed with the Muse Ki67 cell proliferation kit, which identified actively proliferating cells based on the expression of Ki67, a nuclear protein present in the active phases of the cell cycle (G1, S, G2, and M phases) and absent in the resting G0 phase. The Muse Ki67 Proliferation Assay was used with the Guava Muse Cell Analyzer according to the manufacturer’s instructions (Luminex Corporation, Austin, USA). Briefly, the cells were stained after fixation and permeabilization procedures, using Hu Ki67 or Hu IgG1 control fluorochrome-conjugated antibodies to distinguish Ki67(+) or Ki67(-) cells, respectively. The software provided the percentage of both Ki67(+) and Ki67(-) cells.

#### P-gp interaction assay

This experiment was carried out as described by Contino et al. with minor modifications [24]. MDCK-MDR1 cells, stably overexpressing P-gp, (30.000 cells per well) were seeded into black CulturePlate96/wells plate with 100 µL medium and allowed to become confluent overnight. Each test compound solubilized in culture medium was added to cell monolayers, reaching the tested final concentrations (ranging from 0.1 to 100 µM). Each 96/wells plate was incubated at 37 °C for 30 min. 100µL of the pro-fluorescent probe Calcein-AM (2.5 µM in Phosphate Buffered Saline, PBS) was added to each well and the plate incubated for 30 min. Each well was washed 3 times with ice cold PBS. Saline buffer was added to each well and the plate was read with Victor3 (PerkinElmer) at the excitation and emission wave lengths of 485 nm and 535 nm, respectively. In these experimental conditions, Calcein cell accumulation in the absence and presence of tested compounds was evaluated and the fluorescence basal level was estimated in untreated cells. In treated wells the increase of fluorescence compared to basal level was measured. EC_50_ values were determined on the fluorescence increase percentage versus log [dose].

#### ROS determination assay

For ROS analysis the procedure described in [26] was adapted. Briefly, 5 × 10^5^ cells were seeded and incubated for 48 h in the presence and absence of 1 µM of the CB2R reference **compounds 1**and **AM630** and the CB2R agonists **CC48**, **Fi9** and **ASF151**. After the incubation, all the samples were incubated for 30 minutes at 37°C in the dark with 50 µM 2’,7’-dichlorofluorescein diacetate, DCF-DA.

Cells were then washed, detached with trypsin/EDTA solution, centrifuged at 12,000 g for 10 min and re-suspended in1mL PBS. At least 10.000 total events were acquired using an Attune Nxt Acoustic Focusing Cytometer (Life Technologies Corporation) equipped with a blue laser (488 nm). Analysis was performed and data were visualized using Attune™ NxT v2.6 Software. The percentage of green fluorescent cells and the green fluorescence median were measured on a mono-parametric histogram based on DCF-DA fluorescence.

#### Autophagy assay

Cells were rinsed with ice-cold lysis buffer (50 mM Tris,10 mM EDTA, 1% v/v Triton-X100), supplemented with protease inhibitor cocktail set III (80 µM aprotinin, 5 mM bestatin, 1.5 mM leupeptin, 1 mM pepstatin; Sigma-Merck, St. Louis, MO), 2 mM phenylmethyl sulfonyl fluoride and 1 mM Na3VO4, then sonicated and centrifuged at 13,000 g for 10 min at 4 °C. 20 µg protein extracts were subjected to SDS-PAGE and probed with the following antibodies: anti-ATG5 (#ab108327, diluted 1:1000), anti-ATG7 (#ab52472, diluted 1:5000), anti-ATG12 (#ab303488, diluted 1:1000), anti-Beclin (#ab210498, diluted 1:1000), anti-p62 (#ab109012, diluted 1:10000), anti-LC3 (#ab192890, diluted 1:2000)(all from Abcam, Cambridge, UK) or anti-β-tubulin antibody (#sc-5274, diluted 1:1000; Santa Cruz Biotechnology Inc.,Santa Cruz, CA, USA), followed by incubation with a peroxidase conjugated secondary antibody (Bio-Rad Laboratories, Hercules, CA). After washing in Tris-buffered saline (TBS)-Tween 0.1% v/v solution, proteins were detected by enhanced chemiluminescence (Bio-Rad Laboratories).Total protein expression was quantified using the ImageJ software (http://rsb.info.nih.gov/ij/). The expression level of each of the investigated protein was normalized to the β-tubulin level.

#### Apoptosis assay

HGC27-S/R and AGS cells were treated with 1–6 µM **AM630**, 1-10µM **CC48**, 1–10 µM **Fi9**, 1–10 µM **ASF151** and 1–10 µM **compound 1** administered for 48 h. After the specified drug treatments, the cells were processed using the Muse Annexin V/Dead Cell Assay Kit (Luminex Corporation, Austin, USA) for quantitative analysis of live, early/ late apoptotic, and dead cells on a Muse Cell Analyzer. Briefly, the assay utilized Annexin V to detect phosphatidylserine on the external membrane of apoptotic cells. The fluorescent signal emitted by dye-conjugated antibodies was detected by flow cytometry technology (Muse Cell Analyzer, Luminex Corporation, Austin, USA). 7-Amino-Actinomycin D (7-AAD) dead cell marker was also used. The cells were then analyzed according to the user’s guide. In addition, HGC27-S/R cells were treated with 4 nM PTX alone or in combination with 6 µM **AM630**, 10 µM **CC48**, **Fi9**, **ASF151** and **1** for the evaluation of apoptotic status based on caspase3/7 activation as well as for the analysis of cell death by determining cellular plasma membrane permeabilization. After 48 h treatments the cells were processed according to the user’s guide and analyzed with the Muse caspase-3/7 kit (Millipore).The assay provides the relative percentage of living, early/late apoptotic or dead cells.

#### Migration assay

HGC27-S/R and AGS cells were grown until confluence. A scratch wound was generated with a pipette tip. After rinsing with medium to remove detached cells, a low serum medium (1% FBS) with 1–6 µM **AM630**, 1-10 µM **CC48**, 1–10 µM **Fi9**, 1–10 µM **ASF151** and 1–10 µM **compound 1** was added. Photographs were taken of each well immediately (T0) and at various times, T1 (9 h), T2 (24 h), and T3 (48 h), using a Leica DMRXA camera (Leica Microsystems, Milan, Italy). The images were analyzed using ImageJ Software (http://rsb.info.nih.gov/ij/). The distance that cells migrated through the scratch area was determined by measuring the wound width at T1, T2 and T3 and subtracting it from the wound width at the start (T0). The relative migration rate was calculated by setting the percentage of migration of the control cells at time T2 equal to 1 and comparing the percentage of migration of the cells after each drug treatment to this value. The results are representative of three independent experiments.

#### Measurement of VEGFA in cell culture medium

The amount of VEGFA secreted in the culture medium by HGC27-S/R and AGS treated cells was measured using a highly sensitive Enzyme-Linked Immunosorbent Assay (ELISA), Quantikine Kit ELISA (R&D Systems, Minneapolis, MN, USA), according to the manufacturer’s instructions. The measured values were normalized to the number of cells.

#### Statistical analysis

GraphPad Prism 5.0 software (La Jolla, CA, USA) was used to evaluate differences between two unmatched groups with the Mann–Whitney nonparametric test. *P* < 0.05 was considered statistically significant. All experiments were performed in triplicate and repeated three times. Data were presented as mean ± standard deviation (SD).

### In vivo experiment

#### Animals

The study was performed in the Test Facility BIOGEM(BiogemS.c.ar.l. Via Camporeale Area P.I.P., Ariano Irpino (AV) Italy), which is authorized to carry out this experimentation according to Ministerial Authorization n° 257I2023-PR and authorized for the use of animals for scientific purposes and regulatory research according to Italian Decree N° 26/2014: authorization N° 08/2023-UT del 23/03/2023. The Test Facility Biogem is GLP certified and the in vivo phase of the study was performed under GLP-like conditions. The animals’ welfare was checked by trained personnel for any clinical sign of disease or other clinical or behavioural abnormalities. Any deviation from normality was recorded. In case of death, a necropsy was performed. The animals were managed according to Directive 2010/63/UE regarding the protection of animals used for experimental or other scientific purposes, enforced by Italian decree n° 26 of March 4, 2014.

EighteenCD1-nude female mice(Charles River Laboratories International, Inc.) were intraperitoneally injected with the NCI-N87 cell line, stable transfected with the luciferase gene (NCl-N87_LUC) at a concentration of 2 × 10^6^/100µL cells. Experimental groups and treatments are described in the table below (Table 1). Two concentrations of the **CC48** molecule, 10 and 20 mg/Kg, were tested in two experimental groups. A third group was treated with the vehicle, consisting of20% DMSO, 10% Cyclodextrin and distilled water.

**Table 1:** Experimental groups and drug treatments schedule

Experimental Group (GP)	Sex	*N*° of animals	ID Animals From-To	Cell line	Treatment	Dose	Route	Schedule
1	F	6	1–6	NCI-N87_LUC	VEHICLE	-	IP	Everyotherday
2	F	6	7–12	NCI-N87_LUC	CC48	10 mg/kg	IP	Everyotherday
3	F	6	13–18	NCI-N87_LUC	CC48	20 mg/kg	IP	Everyotherday

**CC48** administrations were carried out by intraperitoneal injection (IP) as reported in Table 1, starting from day 5 of the study, considering an administration volume of 10 mL/kg. All the animals were fed ad libitum with complete mouse feed, and water was provided ad libitum. The body weight for each animal was recorded weekly, starting from day 0 until the end of the study. Tumor growth was evaluated weekly using the IVIS Spectrum (PerkinElmer) in vivo technology system, through intraperitoneal injection of D-Luciferin potassium salt (100µL/10 g, PerkinElmer. One animal from each group was excluded from the statistical analysis (see Supplementary Tables S2 and S3): those marked with ID: 1, 7 and 13. According to the ROUT test, these animals were defined as outliers based on the values acquired at the beginning (day 5) and end (day 41) of the measurements. Anomalous peaks (spikes) observed in the data (days 21, 28, and 35) were also excluded from the statistical analysis, as they did not represent real events and could have compromised the integrity of the final results (Table S2).

Physical observations of health status, including visual checks on the mice general appearance (e.g. fur and activity levels/responsiveness), were made daily. On day 45, the animals were individually housed in metabolic cages for 24 h, with free access to water and food, in order to perform urine collection and urinalysis. Immediately before metabolic cage housing, animals were subjected to a water load (10 mL/kg by oral gavage) in order to allow urine volume normalization. In addition, blood samples were taken from the submandibular vein after isoflurane anesthesia and before sacrifice at the end of the study period. The blood was collected in Eppendorf tubes and then centrifuged at about 8,000 rpm for 15 min at 4 °C for serum collection. Mice were sacrificed by CO_2_ and tumor sampling was performed. The tumor masses, when possible and when present, were divided into two halves, one frozen at -80° and the second stored in 10% buffered formalin.

#### Raw data recording and statistical analysis

The staff involved in the study recorded all the observations on recording forms. The data concerning animals’ body weight, clinical signs, feed intake and TP intake were recorded on the experimental register. All the corrections on data collection forms were made according to the Test Facility SOPs. All derived values, of registered tumor volumes, which are reported in the Tables S2 and S3 and relative graphs, such as mean and standard deviation, represent the rounded-up results of calculation performed with Microsoft Excel. Dunnett’s multiple comparison statistical test was applied.

#### Immunohistochemistry

Tissue blocks fixed in formalin and embedded in paraffin were obtained from the explants of the tumor masses of each specimen. Sections stained with hematoxylin and eosin (H&E) were reviewed by a pathologist to confirm the adequacy of the sample, and to evaluate the morphologic and/or pathological characteristics of each sample.

For immunohistochemistry (IHC) detection, 4 μm tumor sections were freshly cut and dried at 60 °C for 30 min. IHC analysis was carried out in sections after deparaffinization for 30 min and then rehydration in grades of alcohol. Antigen retrieval was performed at 90 °C for 20 min with Tris-borate-EDTA Buffer. To assess the Ki67, IHC was again carried out on all cases on paraffin-embedded sections using an anti-Ki67 Ab diluted 1:100 (Mib-1; DAKO) following the manufacturer’s instructions. All cases were reviewed and re-evaluated and then assigned a precise proliferation index number (modal percentage value) that encompassed the amplitude limit of the 2010/2017 WHO range.

#### Quantification of cytokine expression

Serum levels of 23 cytokines [G-CSF, GM-CSF, GRO/KC (CXCL1), IFN-γ, IL-1α, IL-1β, IL-2, IL-3, IL-4, IL-5, IL-6, IL-9, IL-10, IL-12 (p70), IL-13, IL-17 A, IL-18, MCP-1 (CCL2), MIP-1α, MIP-1β, RANTES, TNF-α, and eotaxin] were measured using Bio-Plex Pro Rat Cytokine 23-Plex™ (#M60009RDPD) magnetic bead-based assays (Bio-Rad Laboratories) on the Bio-Plex^®^ platform (Bio-Rad), according to the manufacturer’s instructions. Serum samples were tested, diluted 1:4, and assayed in triplicate. Cytokine expression (pg/mL) for each sample was multiplied by the dilution factor prior to the percent baseline calculation.

## Results

### Cannabinoid receptors subtype 1 and 2 expression levels in different gastric cell models

Expression levels of CB1R and CB2R were studied at both mRNA and protein levels in PTX-sensitive GC lines (HGC27-S, KATO III, AGS, NCl-N87) and in a PTX-resistant cell clone (HGC27-R) derived from the sensitive counterpart, as described in the Methods section. As shown in Supplementary Fig. 1, mRNA of CB1R was highly expressed in HGC27-R, mildly expressed in AGS and HGC27-S cells and poorly expressed in the other cell lines. CB2R was moderately expressed in AGS and HGC27-S and poorly expressed in the other cell lines. Comparable levels of CB1R protein were detected in the different human GC lines, with a slightly higher expression in PTX-resistant cells. CB2R protein levels were higher in HGC27-S/R, moderately expressed in KATO III and NCl-N87, and low in AGS. Therefore, the PTX-sensitive and PTX-resistant lines HGC27 (with high CB2R at both mRNA and protein levels) and AGS line (characterized by the lowest levels of CB2R protein) were used as experimental cell models to investigate the role of CB2R in the growth and spread of human GC and possible correlations with PTX-mediated resistance.

### FAAH inhibitory activity of the selected compounds

To further investigate the involvement of CB2R in GC growth and progression, CB2R ligands were selected including agonist **1** and antagonist **AM630** and three CB2R agonists, **CC48**, **Fi9** and **ASF151**, from our CB2R ligands library. Table 2 shows the affinity and activity profile for both CB1R and CB2R receptors for each compound [16, 19–22]. Furthermore, previous studies have revealed that **CC48** and **Fi9** were also characterized by the ability to induce the endocannabinoid tone by inhibiting Fatty Acid Amide Hydrolase (FAAH), the enzyme responsible for the degradation of both anandamide and related amidated signaling lipids [21]. The present study extended the FAAH inhibitory activity analysis to all the tested compounds **1**, **AM630** and **ASF151**. As expected, compound **ASF151**, bearing an *N*-adamantyl-antranil-amide scaffold similar to that of the dual drug series, presented a FAAH inhibitory profile (IC_50_ = 6.4 µM) comparable to that of **CC48** (IC_50_ = 4.0 µM) and **Fi9** (IC_50_ = 3.4 µM). By contrast, low FAAH inhibition activity was detected for **AM630** and **compound1**.

**Table 2:** Pharmacodynamic profile of the CB2R ligands

Compound	CB2R affinity K_i_, nM ± SEM	CB1R affinity K_i_, nM ± SEM or % at 1µM	CB2R activity EC_50_, nM	CB1R activity EC_50_, nM	FAAHi IC_50_, µM ± SEM
CC48	14.8 ± 4.49^22^	241.3 ± 2.4^22^	123.6^22^	489^22^	4.0 ± 2.3^22^
Fi9	20.1 ± 3.7^22^	67.6 ± 10.0^22^	283.3^22^	252.5^22^	3.4 ± 0.4^22^
ASF151	55.7 ± 10.2^23^	31%^23^	560^23^	-	6.4 ± 1.2
AM630	31.2^21^	-	0.9^20^	-	20 ± 2.8
1	13.4 ± 1.2^17^	-	24.8^17^	-	26.4 ± 7.3

^17,20–23^% at1µM: percentage of displacement at 1 µM; Inhibitor constant (*K*_i_); The Standard Error of the Mean (SEM); half maximal (50) Effective Concentration (EC_50_); half maximal (50) Inhibitory Concentration (IC_50_)

### CB2R compounds cytotoxicity in GC cell lines

**Fig. 1:**
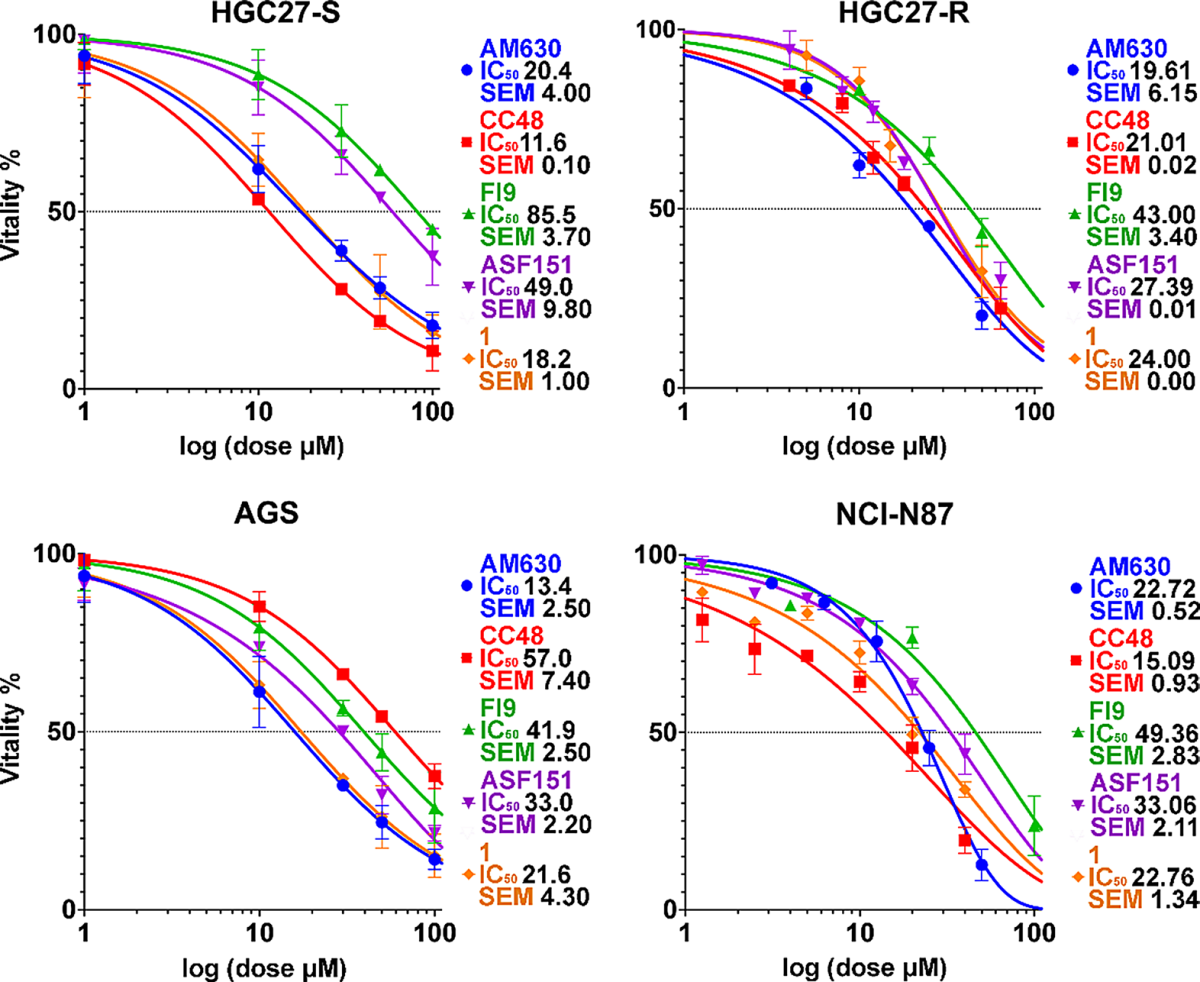
Dose-response curves and IC₅₀ determination of CB2R compounds across gastric cancer cell lines. MTT assays were performed at 48 and 72 h to calculate IC₅₀ values. The graphs represented the results expressed as % of vitality at 48 h and displayed five coloured sigmoid curves, each corresponding to a different CB2R compound: blue for the antagonist **AM630**, red for **CC48**, green for **Fi9**, purple for **ASF151** and yellow for the reference **compound 1**. The corresponding IC₅₀ values with their standard error of the mean (SEM), are shown alongside. Data are from one of three independent experiments; each performed in triplicate

All the CB2R ligands **AM630**, **CC48**, **Fi9**, **ASF151** and **1** were tested for their cytotoxicity at 48 h and 72 h by MTT assay in HGC27-S/R, AGS and NCl-N87 cell lines. The results, in terms of IC_50_, are reported in Table S1. Figure 1 showed the 48-hours dose-response curves of each compound for each cell lines tested, reporting the percentage of cell vitality. The figure also presented the IC₅₀ values, which, together with the values obtained at 72 h, are reported in Table S1. In addition, a statistical analysis was performed to evaluate the significance of differences in IC₅₀ values among different compounds within a given cell line, and of a given compound across different cell lines. The results are shown in the corresponding graphs in Supplementary Fig. 2. Only the differences at 48 h are shown, as all experiments reported in this study were conducted within this timeframe.

In HGC27-S cells, expressing higher levels of CB2R than CB1R, **CC48** exhibited a significantly higher cytotoxicity (IC_50_ value = 11.6 µM) than **Fi9** (IC_50_ value = 85.5 µM) and **ASF151** (IC_50_ value = 49 µM), similar to the reference **Compound 1** (IC_50_ value = 18.2 µM) and **AM630** (IC_50_ value = 20.4 µM).

In HGC27-R cells, the differences in IC₅₀ values were less pronounced, but the trend is similar to that observed in sensitive cells in the presence of the **AM630** antagonist, which has a slightly higher cytotoxicity (IC_50_ value = 13.4 µM) than **CC48** (IC_50_ value = 21 µM), **Compound 1** (IC_50_ value = 21.6 µM), and **ASF151** (IC_50_ value = 27.39 µM). **Fi9** also exhibited a notably higher IC_50_ value (IC_50_ value = 43 µM) in this cell line.

In AGS cells the two CB2R reference compounds **AM630** and **1** showed IC_50_ values of 13.4 µM and 21.6 µM respectively, significantly lower than **CC48** (IC_50_ value = 57 µM), **Fi9** (IC_50_ value = 41.9 µM) and **ASF151** (IC_50_ value = 33.0 µM). However, the IC_50_ values for **CC48** and **Fi9** decrease significantly at 72 h (IC_50_ = 9.8 µM and 7.5 µM, respectively), reaching levels comparable to those of the two reference compounds.

In NCl-N87 cells, the trends in the dose-response curve became comparable to those in the HGC27. The IC_50_ value for **CC48** (IC_50_ value = 15.09 µM) was slightly but significantly lower than for **AM630** (IC_50_ value = 22.72 µM) and **Compound 1** (IC_50_ value = 22.76 µM), and differs further from those for **Fi9** (IC_50_ value = 49.36 µM) and **ASF151** (IC_50_ value = 33.06 µM).

The comparison clearly showed that the dual compound **CC48** tended to have lower IC_50_ values than the other compounds in all lines, except for AGS, which had lower CB2 expression levels. The data in HGC27-S and N87 were particularly interesting because the differences became significant (Fig. S2 panel A). Moreover, analyses of the differences in IC₅₀ values for each compound in different cell lines (Fig. S2 panel B) did not show a correlation between receptor expression levels and IC₅₀ values. However, results for **CC48** showed that lines with higher CB2R levels had lower IC50 values than those found in AGS with lower receptor expression levels. In all experiments performed in this study, each compound was tested at two concentrations: 1 µM concentration to minimize the effects of compounds with a dual target activity on FAAH; 10 µM concentration to test the effects due to the dual activity of **CC48**, **Fi9** and **ASF151** on CB2R and FAAH. The cytotoxicity of all tested compounds was limited at either concentration, being below the IC_50_.

### CB2R compounds inhibit major signaling pathways involved in cell proliferation in GC cells

**Fig. 2:**
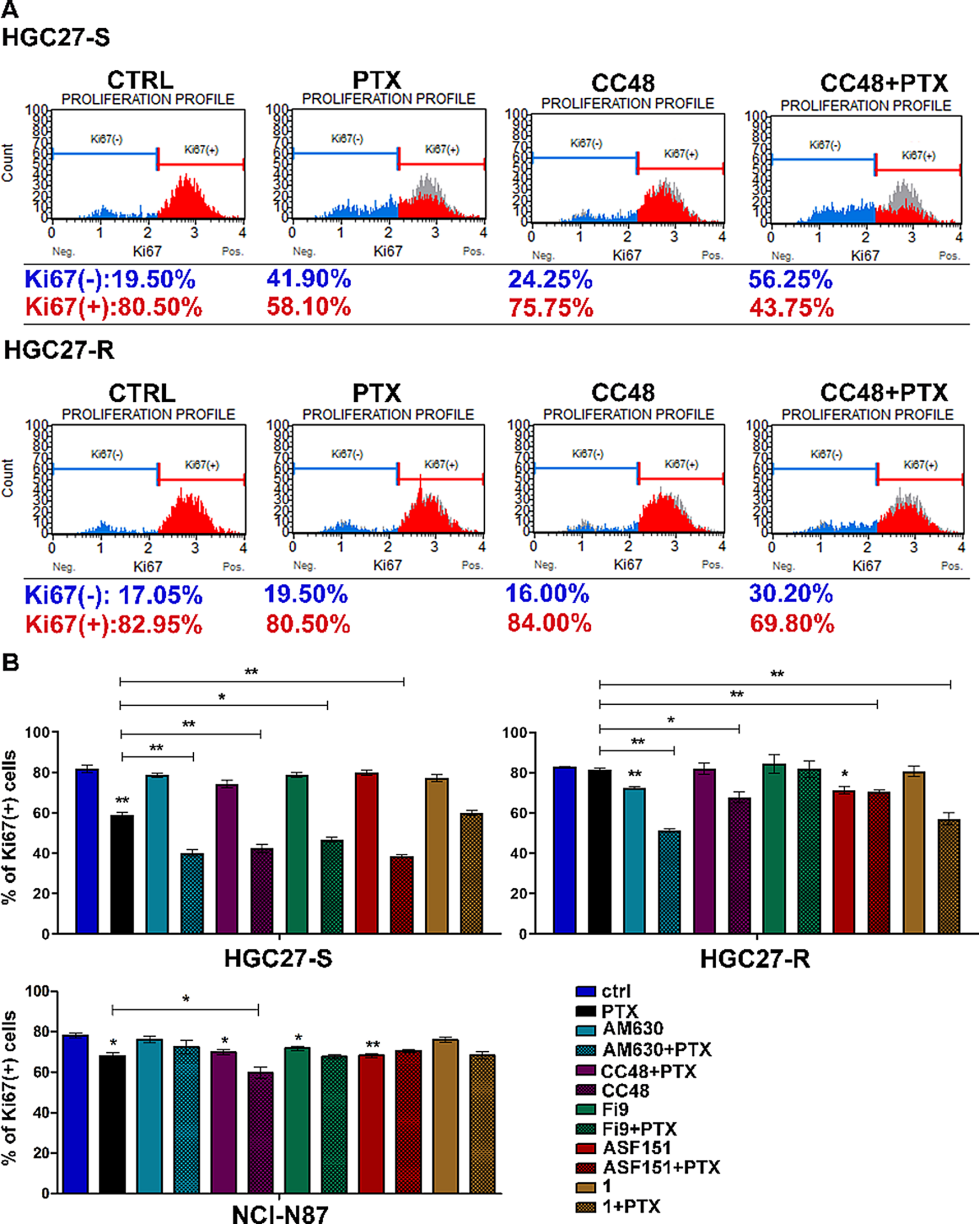
CB2R-binding compounds counter PTX resistance and promote PTX-mediated inhibition of cell proliferation.The Ki67 cytofluorimetric assay was performed after treatment of PTX-sensitive HGC27 cells and their resistant counterparts and NCI-N87 cells with a combination of 10µM **CC48** or **Fi9** or **ASF151** or **1** or 6 µM **AM630** with 4 µM PTX. **A**) Representative flow cytometry charts after exposure to **CC48** and reporting the percentage of Ki67 negative (blue) and positive (red) cells; the Ki67 + population of the control is shown in grey. **B**) Statistical charts reporting the results obtained in HGC27-S, HGC27-R and NCI-N87, from three independent experiments and expressed as means ± SD. Statistical analysis was assessed by comparing the values obtained using single drug treatment to those of corresponding untreated cells and the combined treatments to those with PTX alone, *p < 0.05; **p < 0.01

The effects on cell proliferation of CB2R agonist and antagonist compounds were assayed on GC models, HGC27 and NCI-N87 cell lines and the PTX-resistant HGC27 line. The percentage of Ki67 positive cells (Ki67+) after 48 h of treatment was evaluated using the Muse Ki67 Proliferation Assay. In order to investigate the possible role of these receptors in the PTX resistance mechanism, Ki67 cytofluorimetric assay was performed after treatment of PTX-sensitive HGC27 cells and their resistant counterparts with a combination of 10 µM **CC48** or **Fi9** or **ASF151** or **1** or 6 µM **AM630** with 4 nM PTX. Representative flow cytometry charts reporting the percentage of Ki67 negative (in blue) and positive (in red) cells and statistical charts reporting results from three independent experiments in HGC27-S, their resistant counterpart and in NCI-N87 are reported in Fig. 2.

In the HGC27-S cells, among the treatments with CB2R-targeted compounds, only **CC48** resulted in a slight decrease in the percentage of Ki67 + cells (75.75%) compared to control cells (80.50%), the percentage of actively proliferating cells decreased dramatically after treatment with 4 nM PTX (58.1%) and this percentage was further significantly reduced with all PTX combination treatments with 10 µM **CC48** (42.3%), 10 µM **Fi9** (46.8%),10 µM**ASF151** (38.6%), 10 µM **compound 1** (60%), and 6 µM **AM630** (40.1%).

In NCI-N87 cells, the percentage of actively proliferating cells (KI67+) significantly decreased following treatment with 2 nM PTX (68.1%) compared to control cells (78.2%). A significant reduction in KI67 + cells was also observed with single-agent treatments using 10 µM **CC48** (60.9%), **Fi9** (71.8%), and **ASF151** (68.3%). Of the combination treatments with PTX, only cotreatment with **CC48** resulted in a further significant decrease in the percentage of proliferating cells (59.9%), compared to PTX alone (68.1%). As expected, in HGC27 PTX-resistant cells, single treatment with this chemotherapy agent did not significantly modify the percentage of proliferating cells (80.5% vs. 82.95%), nor did single treatments with CB2R compounds. Surprisingly, combined treatments of PTX and 10 µM **CC48**, 10 µM **compound 1 **or 6 µM **AM630** resulted in a significant reduction in Ki67-positive cells with percentages falling to 67.9%, 57.1% and 51.3% respectively. In order to assess the possible contribution of the interaction of the tested compounds with P-gp, the main pump belonging to the MultiDrug Resistance (MDR) proteins family, we tested compounds for their ability to modulate P-gp, given that a previous study [27] identified the efflux protein P-glycoprotein (P-gp) as one of the main factors of PTX resistance in HGC27-R cells. The data shown in Table 3 excluded interaction with P-gp for compounds **Fi9** and **ASF151**, while compounds **AM630** and **compound 1** showed a low interaction with the pump with respect to the P-gp reference compound verapamil [28]. **CC48** was the only compound to show a moderate interaction with P-gp, suggesting a potential ability to overcome MDR in resistant cancer cells through this interaction.

**Table 3:** CB2R ligands interaction with P-gp

Compound	*P*-gp activity, EC_50_,µM ± SEM^a^
CC48	7.75 ± 1.25
Fi9	> 100µM
ASF151	> 100µM
AM630	16.8 ± 3.20
1	24.7 ± 3.82
verapamil	0.5 ± 0.1

^a^ Values are the mean ± SEM of three independent experiments performed in triplicate; half maximal (50) Effective Concentration (EC_50_); The Standard Error of the Mean (SEM)

**Fig. 3:**
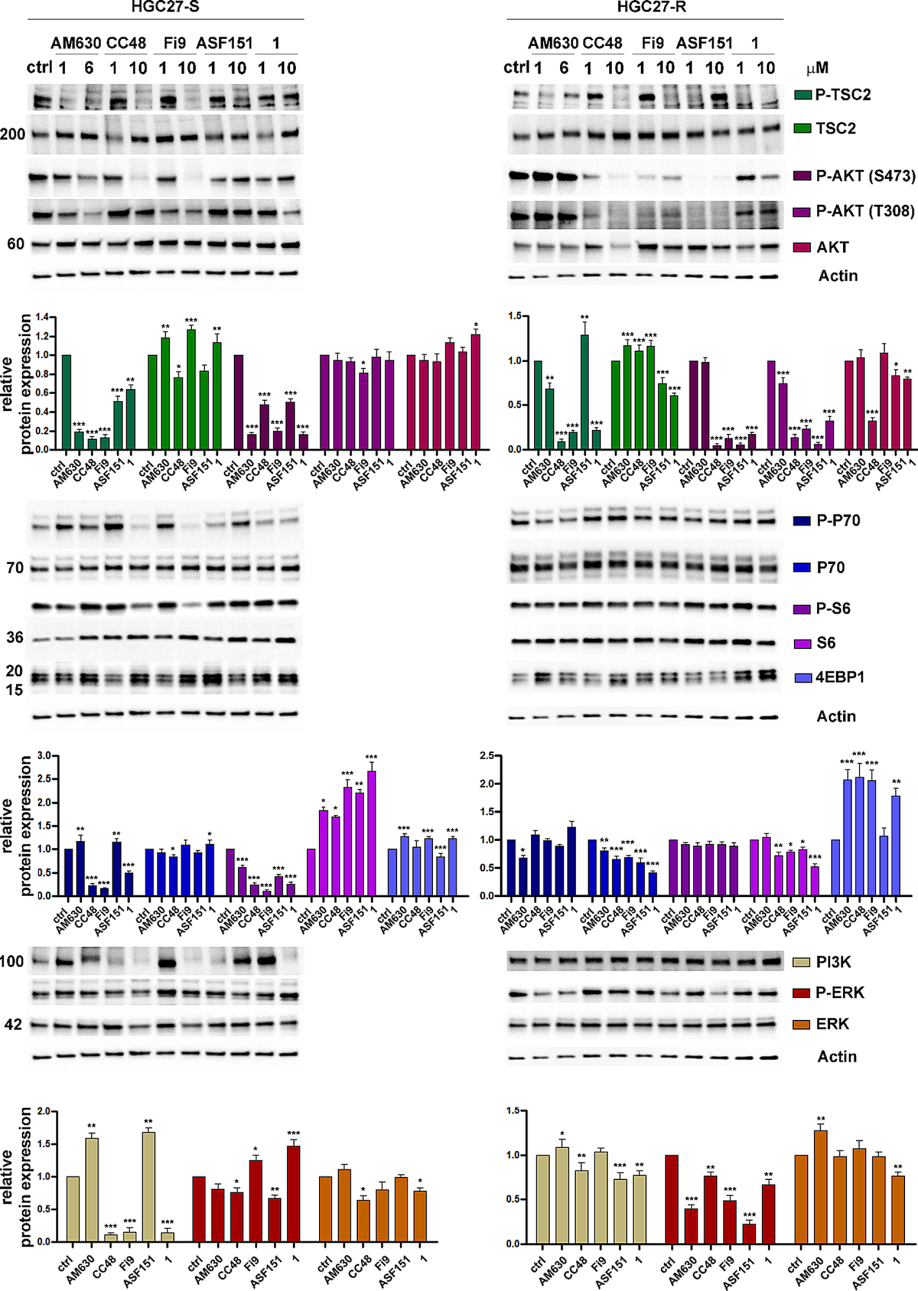
Western blot analysis of the expression and activation levels of key proteins involved in cell proliferation. Representative western blotting analyses performed in HGC27-S/R and statistical charts reporting the results from three independent experiments expressed as means ± SD. The expression of the phosphorylated and/or total forms of TSC2, AKT, p70, S6, 4EBP1, PI3K, and ERK1/2 after 48 h of treatment with the compounds **AM630**, **CC48**, **Fi9**, **ASF151 **and **1**. The expression levels of each of the investigated proteins were normalized to the actin level, *p < 0.05; **p < 0.01; ***p < 0.001

The anti-proliferative effects of CB2R agonist and antagonist compounds at both concentrations tested, were also assessed by WB assay after 48 h of treatment. The analysis was extended to some of the major proteins involved in key signaling pathways regulating cell proliferation, such as PI3K/Akt/mTOR and ERK1/2. For each of the investigated proteins, the expression of the functionally active/inactive form was determined by analyzing specific phosphorylation sites, together with the expression of its overall levels. We selected two different concentrations, as above mentioned, 1 and 10 µM, for the CB2R agonists **1**, **CC48**, **Fi9** and **ASF151** and 1 µM and 6 µM (concentrations below the IC_50_ in all lines analyzed) for the antagonist **AM630**. The results of representing WB for HGC27-S/R and the graphs for three independent experiments are shown in Fig. 3. Corresponding results from AGS experiments are shown in Figure S3. In HGC27-S cells, both the antagonist 6 µM **AM630** and the agonist compounds 10 µM **CC48**/**Fi9** determined a significant decrease in PI3K protein expression with a consequent decrease of the activated Akt (phosphorylation in T308 and Ser473), which, through the inhibitory phosphorylation (S9) of TSC2, a key repressor of mTORC1, resulted in both an increased inhibition of p70S6K and its target S6 involved in protein synthesis, as well as an increased expression of the inhibitory protein 4EBP1, thus inhibiting the translation machinery.

The inhibitory effects induced by the reference agonist **1** were not detected for all target proteins, whereas no effect was detected after treatment with **ASF151**. Inhibitory effects on ERK1/2 were slight and significant only with 10 µM **CC48**. As reported in the literature [20], due to the possible interaction on specific molecular targets other than CB2R, **AM630** was observed to exert agonist ligand-like effects on some of the pathways studied. In PTX-sensitive AGS cells, treatment with the 10 µM **CC48** agonist significantly inhibited proliferation through substantial changes in the expression of all molecules investigated. Minor effects were observed after treatment with the other agonist molecules and with the **AM630** antagonist at both concentrations. In HGC27-R cells, as compared with the sensitive counterpart, the inhibitory effects of **CC48** and **Fi9** agonists remained strong and significant for the TSC2, Akt and ERK1/2 targets, but slight or absent for the other proteins investigated. In these cells, similar effects were detected with 10 µM **ASF151** and **compound1**. With the antagonist **AM630** the inhibitory effects on TSC2, P70 and ERK1/2 persisted, while no effects on PI3K, Akt, S6 and 4EBP1 were seen.

### CB2R compounds differently impact ROS production in GC cells

**Fig. 4:**
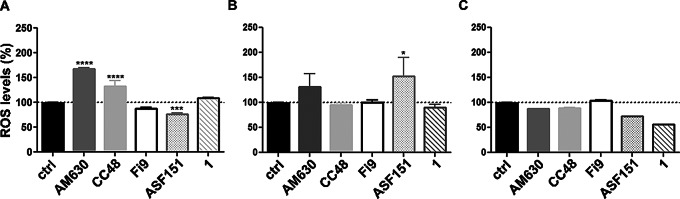
Reactive Oxygen Species (ROS) induction. Production of the Reactive Oxygen Species (ROS) as percentage vs. untreated cells (ctrl) after 48 h treatment with each CB2R ligand at 1 µM in HGC27-S cells (**A**), in HGC27-R cells (**B**), in AGS cells (**C**). Data are means *±* SD (*n* = 3). Significant results are marked as follows: *p < 0.05; ***p < 0.001; ****p < 0.0001

All the compounds were also tested for their ability to induce reactive oxygen species (ROS) production as oxidative stress triggers in all the cell lines studied. Preliminary experiments were carried out to set the experimental conditions. The best compromise was reached at 48 h treatment and a 1 µM concentration for all ligands. At 72 h and at 10 µM, the ROS basal level was too high to appreciate significant differences. In the HGC27-S cell line, the two CB2R compounds **AM630** and **1**, and the CB2R agonist **CC48**, induced a significant increase in intracellular ROS production (Fig. 4A). In the HGC27-R cells, only the CB2R antagonist **AM630** and the CB2R selective agonist **ASF151 **induced an increase in ROS production (Fig. 4B). In AGS cells, all compounds were unable to induce ROS production, as depicted in Fig. 4C.

Due to the possible agonist-like effect on specific molecular pathways, ROS induction was observed in both HGC27 cell lines following treatment with the compound **AM630**. This effect, similar to that induced by some of our tested CB2R agonists, is likely attributable to interactions with other molecular targets [20].

### CB2R compounds induce autophagy in paclitaxel-resistant GC cells

**Fig. 5:**
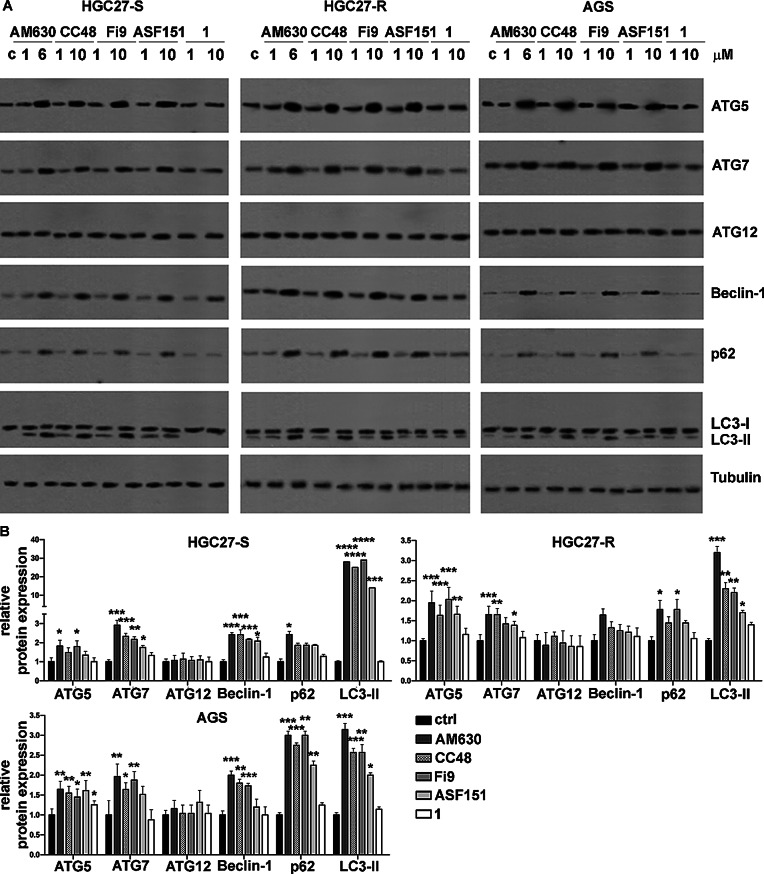
Western blot analysis of expression levels of key proteins involved in autophagy. **A)** Representative WB experiments showing the expression levels of autophagy-involved proteins ATG5, ATG7, ATG12, Beclin-1, LC3-II in PTX-sensitive and resistant HGC27 cell lines and AGS after 48 h of treatment with the compounds **AM630**, **CC48**, **Fi9**, **ASF151** and **1**. **B**) Statistical charts reporting the results from three independent experiments and expressed as means ± SD. Expression levels of each of the investigated proteins were normalized to the Tubulin level, *p < 0.05; **p < 0.01; ***p < 0.001;****p < 0.0001

The effects on autophagy of the CB2R agonist and antagonist compounds were assayed on the GC, HGC27-S/R and AGS cell lines. The effects of each compound at both concentrations tested were assessed after 48 h by WB determination; results are shown in Fig. 5. Untreated HGC27-R cells showed a higher basal level of autophagic markers, such as ATG5, ATG7, ATG12, Beclin-1, LC3-II, compared to their sensitive counterpart HGC27-S. Untreated AGS showed an intermediate level of autophagic markers between HGC27-R and HGC27-S cells. When treated with the reference CB2R antagonist **AM360**, in all cell lines we obtained a dose-dependent increase of autophagic proteins, except in ATG12. A similar trend was obtained with the two multi-target agents **CC48** and **Fi9**; **ASF151** was the less potent inducer of some autophagy-related proteins such as p62 and LC3-II. By contrast, the pure CB2R agonist **1** did not change the expression of autophagy proteins. The increase in ATG5, ATG7, beclin-1, p62 and LC3-II induced by CB2R ligands was more pronounced in HGC27-R than in HGC27-S cells, while it was slight in AGS cells.

### CB2R compounds induce apoptosis in paclitaxel-resistant GC cells

**Fig. 6:**
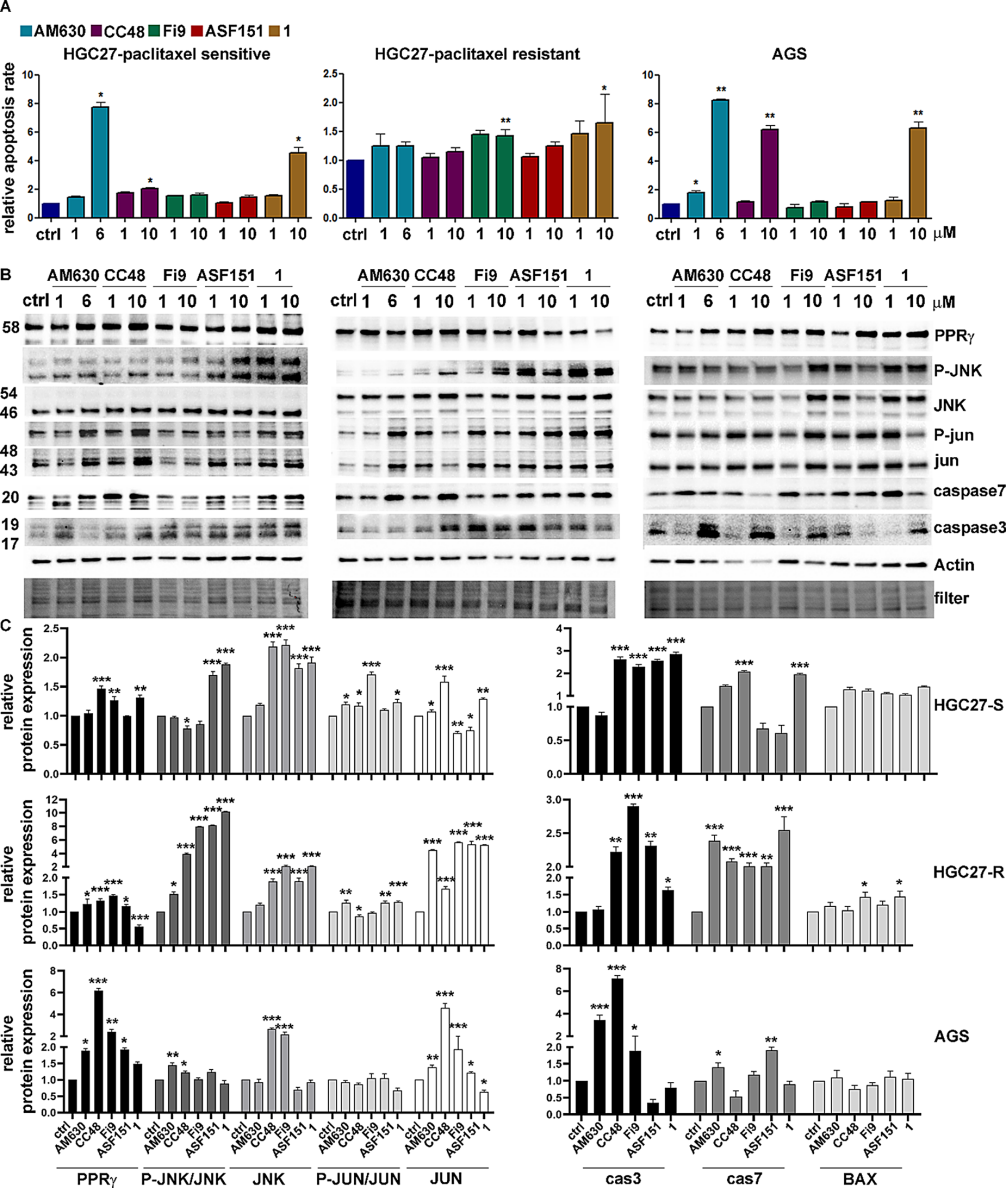
Apoptotic effects of CB2R ligands in GC cell lines. **A**) Muse Annexin V Cell Assay for HGC27-S/R and AGS cell lines evaluated after 48 h of treatment with 1 µM or 6 µM of **AM630** and 1 µM or 10 µM of **CC48**, **Fi9**, **ASF151 **and **compound 1**. Results expressed as relative apoptosis rate compared to control cells and derived from three independent experiments were expressed as mean ± SD and plotted in the corresponding graphs. *p < 0.05; **p < 0.01; **B**) Representative western blotting analyses performed in HGC27-S/R and AGS cells regarding the expression of PPRγ, P-JUN/JUN, P-JNK/JNK and cleaved caspase 3/7. Actin was used as a normalizer of the protein extracts. **C**) Statistical charts reporting the results from three independent western blotting experiments performed in HGC27-S/R and AGS cells and expressed as means ± SD, *p < 0.05; **p < 0.01; ***p < 0.001

The effects on apoptosis of the CB2R agonist and antagonist compounds were assayed in the HGC27-S/R and AGS cell lines. The cells were incubated with drugs and 48 h later, the effects on apoptosis were evaluated using the Annexin V cytofluorimetric assay. In HGC27-S cells, all drugs at the concentration of 1 µM resulted in only a slight increase in the percentage of apoptotic cells compared with the percentage of apoptosis in control cells. Nevertheless, concentrations of 6 µM **AM630**, 10 µM of **CC48** and 10 µM of **compound 1** significantly enhanced the pro-apoptotic effect; the increases observed with these molecules were 8-, 2- and 4.5-fold, respectively, compared to untreated cells. The results obtained in AGS cells with the same compounds and in the same experimental conditions are similar to findings in HGC27-S cells; a significant induction of apoptosis was also reported in these cells by 6 µM **AM630**, 10 µM **CC48**, and 10 µM **compound 1**, with 8-, 6-, and 6-fold increases, respectively. In HGC27 cells resistant to PTX, although the inductive effects on apoptosis by the tested compounds were less intense than those observed in sensitive cells, these effects are still significant with 10µM **Fi9 **and 10 µM **compound 1**, showing increases of 1.4 and 1.7, respectively. The results are shown in the graphs reported in panel A of Fig. 6.

**Fig. 7:**
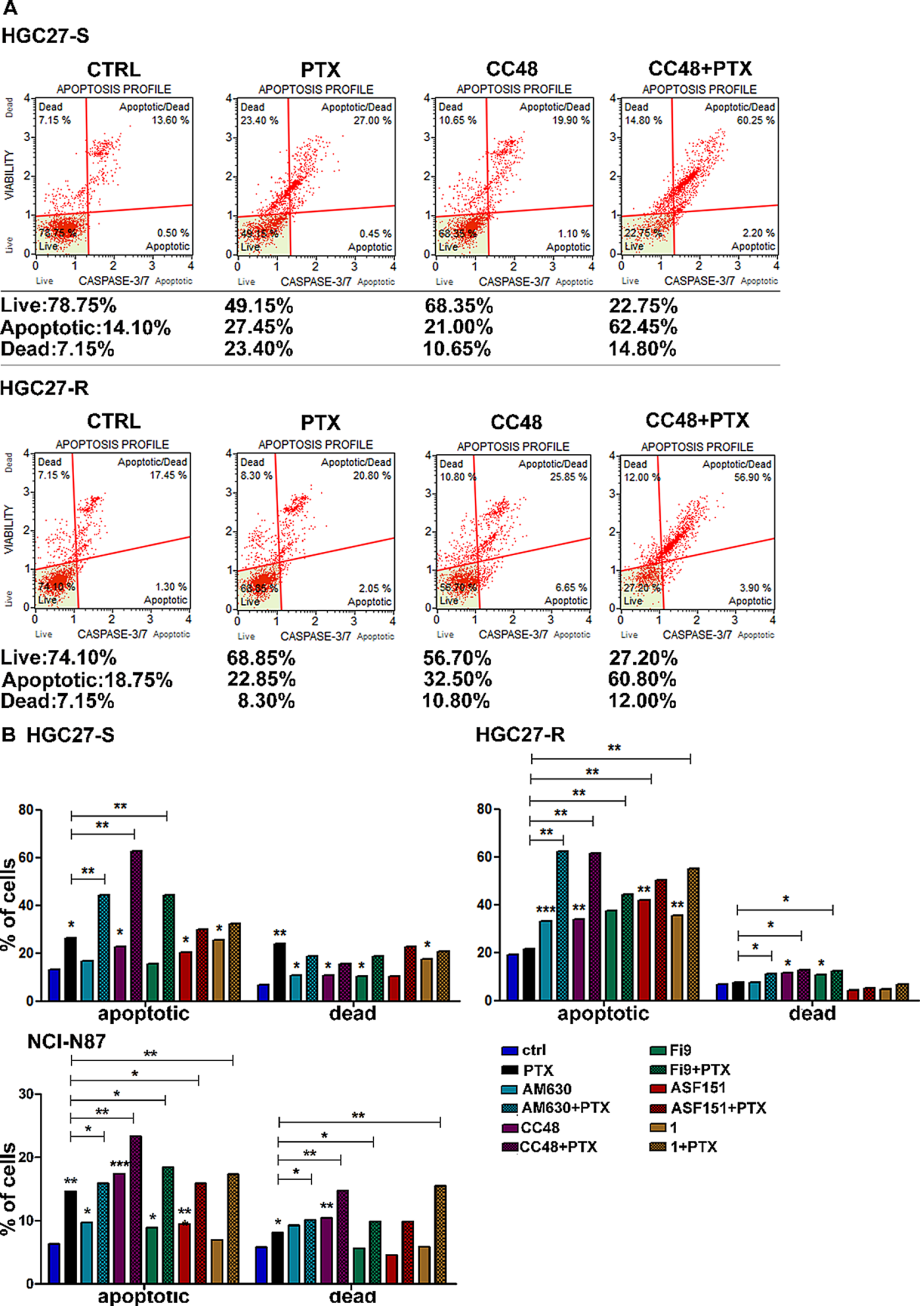
Apoptotic profile of CB2R ligands and PTX in HGC27-S/R and NCI-N87 cells. The Muse caspase 3/7 activation Cell Assay was assessed after 48 h of both single drug treatments with 4 nM PTX, 6 µM **AM630**, 10 µM **CC48**, **Fi9**, **ASF151** and **compound 1**, and after combined treatment of PTX with CB2R target compounds. **A**) Representative flow cytometry charts of HGC27-S/R are shown. Four cell populations can be distinguished, namely the percentage of live cells (bottom left quadrant), cells in early apoptosis (bottom right quadrant), in late apoptosis (top right quadrant) and dead cells (top left quadrant). **B**) The results derived from three independent experiments on HGC27-S, HGC27-R and NCI-N87 cell lines are expressed as means ± SD and reported in the relative graphs. Statistical analysis was conducted, comparing the values obtained using single drug treatment to those of corresponding untreated cells and the combined treatments to those of the single treatments, *p < 0.05; **p < 0.01; ***p < 0.001

In addition, the expression of proteins actively involved in inducing apoptosis was investigated both in HGC27-S/R and AGS cells; results are shown in panel B of Fig. 6. In both HCG27-S/R and AGS cells, CB2R antagonist and the agonists investigated caused a strong activation of the pro-apoptotic cascade JNK/JUN as well as the activation of both PPRγ and caspase 3/7, revealed in their cleaved active form. The investigated markers were significantly induced in HGC27-S and AGS cells. The compounds with the greatest inductive effects at the highest concentration (10 µM) were **CC48** and **1** in the HGC27-S cell line and **CC48**, **Fi9** and **1** in AGS cells. In HGC27-R cells, although the pro-apoptotic action of CB2R agonists was less robust and showed some differences among the markers involved, it was still significant. Although in resistant cells the pro-apoptotic action of **AM630 **was not detected in any of the proteins investigated, a significant activation of JUN and caspase 7 was found. These results were further supported by cytofluorimetric analysis of caspase 3/7 activation in HGC27-S/R and NCI-N87 samples treated with CB2R-targeted compounds in combination with PTX. Caspase 3/7 activation was assayed using the Muse caspase 3/7 activation kit, in the same experimental conditions as previously described, and after combined treatment of the targeted CB2R compounds with 4 nM PTX in both HGC27-S/R lines and with 2 nM PTX in NCI-N87 line. Representative flow cytometry charts of HGC27-S/R reporting the percentage of live, apoptotic, apoptotic/dead and dead cells and statistical charts of HGC27-S/R and NCI-N87 reporting results from three independent experiments are showed in Fig. 7.

In HGC27 PTX-sensitive cells, PTX treatment did not only significantly increase caspase 3/7 activation compared to untreated cells (26.2% vs. 13.05%),but also increased cell death (23.7% vs. 6.6%). Treatment with the antagonist **AM630** (6 µM) resulted in only a slight increase in the activation rate of caspase 3/7 and dead cells, but the combination with PTX resulted in a further increase in apoptotic cells compared to PTX treatment alone (44.3% vs. 26.2%), without leading to an increase in toxicity. The agonist compound **CC48** (10 µM) caused a slight but significant increase in the percentage of apoptotic cells compared to control cells (21% vs. 14.1%) without affecting the number of dead cells, and the combination treatment resulted in a further increase in apoptotic cells compared to PTX treatment (62.7% vs. 26.2%). The compound **Fi9** (10 µM) increased the apoptotic induction exerted by PTX in combined treatments (44.45% vs. 26.2%). **ASF151** (10 µM) and **compound 1** had a significant effect on increasing the number of apoptotic cells (20.25% vs. 13.05% and 25.4% vs. 13.05%, respectively) compared to untreated cells after the single treatments, but no further increase over PTX in the combination treatments. In resistant cells, as expected, PTX produced only a small pro-apoptotic effect (21.4% vs. 18.9%) and did not induce cell death (7.70% vs. 6.6%). Although targeted CB2R compounds all had pro-apoptotic effects respect to untreated cells, with increases by 32.9% vs. 18.9% for **AM630**, 33.8% vs. 18.9% for **CC48**, 37.5% vs. 18.9% for **Fi9**, 42% vs. 18.9% for **ASF151** and 35.3% vs. 18.9% for **compound 1**. The combination of each of these compounds with PTX resulted in a further significant increase in apoptotic cells to a percentage of 62.4% (**AM630**), 61.4% (**CC48**), 44.2% (**Fi9**), 50.4% (**ASF151**) and 55.2% (**compound 1**), without significant effects on cytotoxicity. In NCI-N87 cells, treatment with PTX alone significantly increased the proportion of cells with activated caspase 3/7 (14.6%) compared to untreated controls (6.35%). PTX treatment also increased the percentage of dead cells compared to the control group (8.1% vs. 5.8%). Similarly, single treatments with 6 µM **AM630** and 10 µM of **CC48**, **Fi9** and **ASF151** all significantly induced caspase 3/7 activation (9.5%, 17.4%, 8.9% and 9.5% respectively vs. 6.35%), with **CC48** demonstrating the most pronounced effect. **CC48** treatment alone also significantly increased cell death related to the control group (10.4% vs. 5.6%). Combining PTX with each CB2R compounds further enhanced caspase 3/7 activation compared to PTX alone, reaching 15.9% with **AM630**, 23.3% with **CC48**, 18.5% with **Fi9**, 15.9% with **ASF151** and 17.3% with **compound 1**. Increased cell death was observed in all combination treatments except for PTX with **ASF151**, in which there was no significant difference compared to PTX alone.

### CB2R compounds inhibit migration in paclitaxel-resistant GC cells

**Fig. 8:**
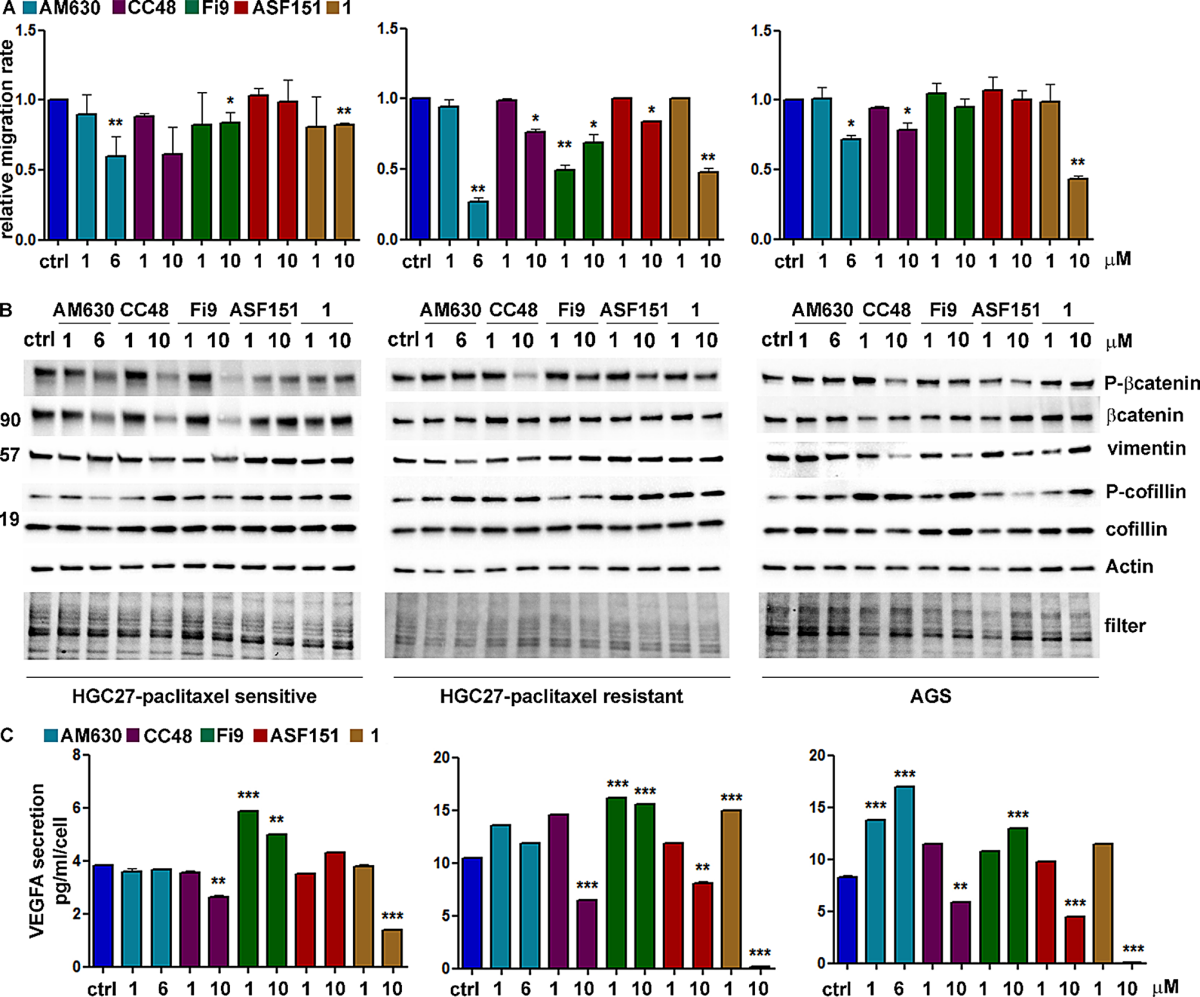
Effects of CB2R ligands on inhibition of cell migration and VEGFA secretion. **A**) Scratch assay evaluated on HGC27-S/R and AGS treated with 1 and 6 µM **AM630** and 1 and 10 µM **CC48**, **Fi9**, **ASF151** and **1**. Cells were microscopically analyzed at the time of scratching (T0) and after 24 h (T1). The relative migration rate was calculated by placing the percentage migration of control cells at time T1 equal to 1 and comparing the percentage migration of cells after each drug treatment with this value. The experiments were performed in triples and the average SD values were plotted in the relative graph. *p < 0,05; **p < 0,01; **B**) Representative western blotting analyses performed in HGC27- S/R and AGS cells regarding the expression of P-βcatenin/βcatenin, vimentin and P-cofillin/cofillin. Actin was used as a normalization of the protein extracts; **C**) Effects of CB2R ligands on VEGFA/VEGFC secretion. The ELISA assay was assessed on HGC27-S/R and AGS treated with 1 and 6 µM **AM630** and 1 and 10 µM **CC48**, **Fi9**, **ASF151** and **1**. The concentration of VEGFA was determined in the medium and normalized for the cell number. The values ± SD, obtained from three independent experiments expressed as pg/mL were shown in the relative graphs.**p < 0,01; ***p < 0,001

The effects on apoptosis of the CB2R agonist and antagonist compounds in HGC27-S/R and AGS cells have also been investigated on cellular motility. In this regard, a scratch assay was performed after treatments with the compounds in the same experimental conditions described above. The results shown in panel A of Fig. 8 revealed that in HGC27-S cells, the migration rate was found to be significantly reduced following treatment with 6 µM **AM630**, 10 µM **Fi9**, and 10 µM **compound 1** compared to untreated cells (where the rate is set equal to 1) with decreases of 0.6 vs. 1, 0.8 vs. 1 and 0.8 vs. 1, respectively. A decreasing trend in migration was also observed with 10 µM **CC48** (0.6 vs. 1); by contrast, no effect was observed with **ASF151** at both concentrations used. Similarly, in AGS cells 6 µM of **AM630**, 10 µM of **CC48**, and 10 µM of **compound 1** caused a drop to 0.7 vs. 1, 0.8 vs. 1 and 0.4 vs. 1, respectively. A lesser effect was observed with **Fi9** and no effect with **ASF151**. In HGC27-R, the inhibitory effects of all test compounds at the highest concentration were robust and significant, the migration rate compared to control cells was 0.2 with compound **AM630**, 0.76 with **CC48**, 0.69 with **Fi9**, 0.8 with **ASF151** and 0.48 with **compound 1**.

The reduction in the rate of migration was also verified by the reduction in the activity of some proteins involved in Wnt/βcatenin signaling resulting in a reduced epithelial-mesenchymal transition (reduced mesenchymal marker vimentin) and migration (increased inhibitory phosphorylation of cofillin). The representative WB for the three lines investigated are shown in panel B of Fig. 8. Again, **AM630**, **CC48** and **Fi9** and **compound 1** were found to be more effective. Contrasting results depending on the marker and cell line used were noted with **ASF151**. Extending the analysis to one of the major factors involved in angiogenesis, secretion in serum-depleted culture medium of VEGFA was investigated after treatments with both the CB2R antagonist and agonist molecules. VEGFA/VEGFC secretion experiments in the culture medium were conducted in HGC27-S/R and AGS cell lines treated with 1 and 6 µM of **AM630** and with 1 and 10µM of **CC48**, **Fi9**, **ASF151** and **compound 1**. The conditioned media were collected after 48 h of drug treatments and the growth factor concentrations were detected using specific ELISA kits, as described in the Methods section. The results obtained are reported on panel C of Fig. 7. In PTX sensitive HGC27-S cells a significant decrease was observed after 10 µM of **CC48** (2.6 pg/mL/cell vs. 3.82 pg/mL/cell) and 10 µM of **compound 1** (1.4 pg/mL/cell vs. 3.82 pg/mL/cell); these two compounds acted similarly in AGS with reductions of 5.8 pg/mL/cell vs. 8.3 pg/mL/cell and 0.1 pg/mL/cell vs. 8.3 pg/mL/cell, respectively. In these cells also 10 µM of **ASF151** had an inhibitory effect, with a VEGFA reduction of 4.4 pg/mL/cell vs. 8.3 pg/mL/cell. In the PTX-resistant HGC27-R cell line the effects of 10 µM **CC48**, **ASF151** and **compound 1** were comparable, showing reductions of 6.5 pg/mL/cell vs. 10.5 pg/mL/cell, 8 pg/mL/cell vs. 10.5 pg/mL/cell and 0.2 pg/mL/cell vs. 10.5 pg/mL/cell. Unexpectedly, both with the antagonist **AM630** and the agonist **Fi9**, an increase in VEGFA secretion resulted in all the cell lines investigated.

### CB2R agonist CC48 reduces tumor masses in mice, with limited hepatic and renal toxicity

**Fig. 9:**
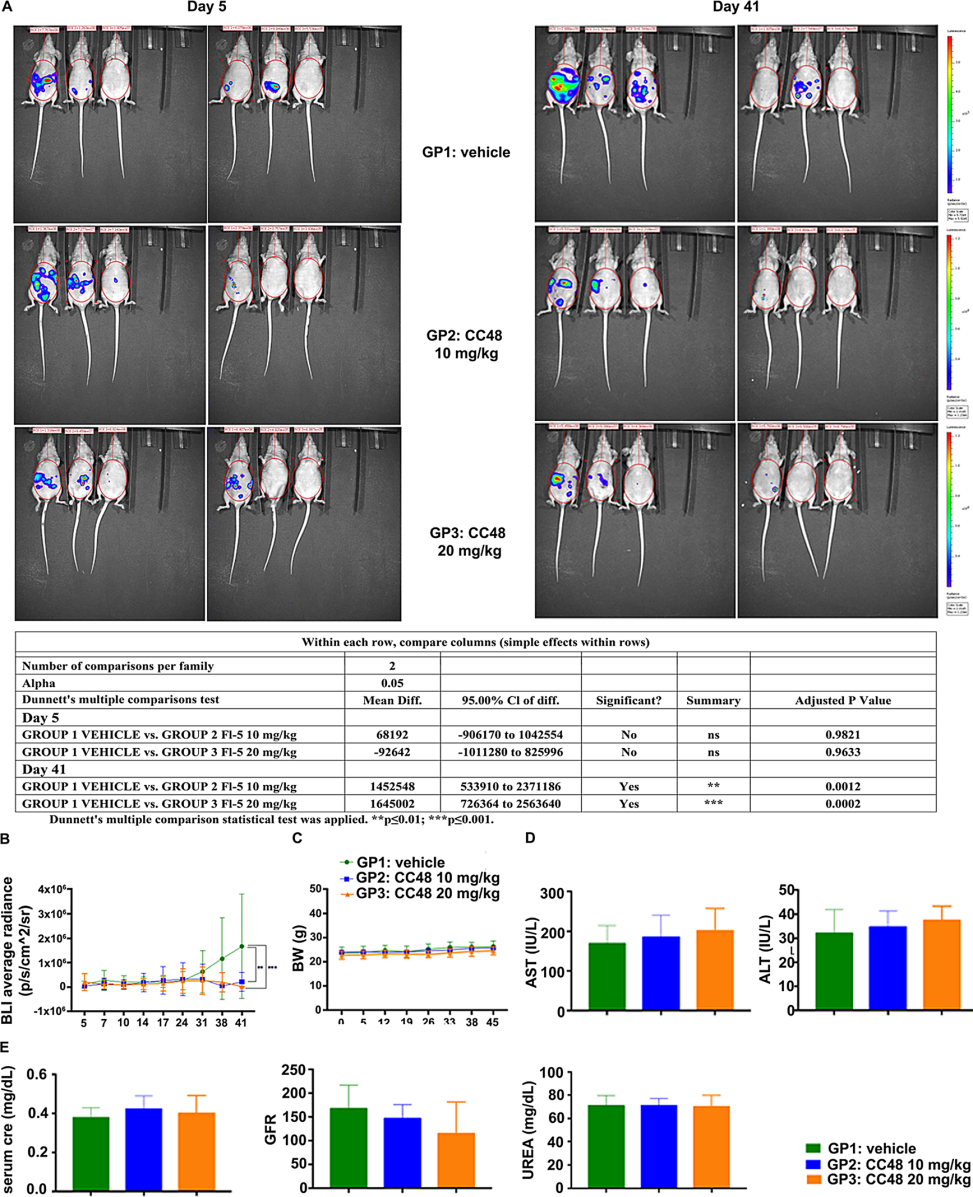
CB2R agonist CC48 reduces tumor masses in mice. **(A)** Tumor growth was evaluated weekly using an IVIS spectrum PerkinElmer. Tumor masses measurements were started on the fifth day after intraperitoneal inoculation of GC cell line NCl-N87_LUC and were performed twice a week until day 41; **(B)** From the statistical analysis performed on the data obtained throughout the study (up to day 41), a substantial and significant decrease in tumor volume was observed at day 41 in mice treated with both **CC48** concentrations (GP2, 10 mg/kg and GP3, 20 mg/kg), compared to the control group (vehicle) (**p < 0.01 for GP2 and ***p < 0.001 for GP3).Dunnett’s multiple comparison statistical test was applied; **(C)** No statistically significant differences were observed between the control group (vehicle) and the two treated groups (GP2 and GP3) in relation to body weight, suggesting that the drug did not negatively affect the growth or energy balance of the animals. **D-E)** Similarly, the biochemical **(D**,** E)** and urinary **(E)** parameters analysed did not show any significant alterations in the treated groups (GP2 and GP3), as compared to the control group. Abbreviations BW: Body Weight; AST: Aspartate Transferase; ALT: Alanine Aminotransferase; Cre: Creatinine; GFR Glomerular Filtration Rate

Compound **CC48** was identified as the best candidate for validation of an anti-tumor activity in the in vivo GC model following evaluation of all the in vitro findings. The efficacy of the **CC48** compound was evaluated in mouse models obtained by intraperitoneal inoculation of NCI-N87_LUC cells, a cell line derived from a well-differentiated human gastric carcinoma [29]. Eighteen nude female mice were intraperitoneally injected with the NCI-N87_LUC cell line, at a concentration of 2 × 10^6^/100 𝜇L cells. The animals were randomized into three experimental groups corresponding to three different treatments (GP1: vehicle, GP2: 10 mg/Kg **CC48**, GP3: 10 mg/Kg **CC48**) as shown in the table in the Methods section. Tumor growth was measured twice a week using an IVIS spectrum. Tumor mass measurements were started on the fifth day after intraperitoneal inoculation of NCl-N87_LUC and were performed twice a week until day 41. The tumor volumes are listed in Table S2 and the acquisition images are presented in panel A of Fig. 9. Statistical analysis (Table S3) performed on the data obtained throughout the study (up to day 41) showed a substantial and significant decrease in tumor volume at day 41 in mice in group 2 treated with the lowest dose of **CC48** (GP2, 10 mg/kg), compared to the control group (vehicle). A statistically significant difference in the size of the tumor mass was also found in mice in group 3 treated with the highest dose of the compound (GP3, 20 mg/kg) compared to the control group (vehicle). The images (Fig. 9A) and corresponding graphs (Fig. 9B) showed that the signals detected by the IVIS 41 days after the start of treatment were higher in the control group than in the **CC48** treated groups (low dose: GP2 and high dose: GP3). This suggests that the **CC48** drug reduces signals compared to the control group. Furthermore, the reduction in signals appears to be more pronounced in the high dose group (GP3), suggesting that the high dose may be more effective in inhibiting tumor growth. Therefore, the significance values (**p < 0.01 for GP2 and ***p < 0.001 for GP3) confirm that the differences between the treated groups and the control group are statistically significant, showing the greatest efficacy in the high dose group. No statistically significant differences were observed between the control group (vehicle) and the two treated groups (GP2 and GP3) in relation to body weight, suggesting that the drug did not negatively affect the growth or energy balance of the animals (Fig. 9C). At the end of the study, the animals that had received the NCI-N87_LUC cells line intraperitoneally were sacrificed by CO_2_ and tumor sampling was performed. No pathological changes were found at the necropsy examination and in some animals no visible and/or removable tumor masses were found. Only the one animal with ID_1 (GP1) presented abdominal ascites, probably caused by the different tumor masses present on different organs. Tumor masses, where possible, were divided into two halves, one frozen at -80° and the second stored in 10% buffered formalin. The sampled tumor masses and some removed organs were photographed on graph paper (data not shown). On day 45, the animals were individually housed in metabolic cages for 24 h, with free access to water and food, in order to perform urine collection and urinalysis. Immediately before metabolic cage housing, animals were subjected to a water load (10 mL/kg by oral gavage) to allow urine volume normalization. Urine samples collected over 24 h were analyzed to calculate the glomerular filtration rate (GFR) reported in Fig. 9E. The results of the serum concentration of key markers of liver (AST and ALT) and kidney (Cre, UREA) function in blood samples collected at the time of animal sacrifice are shown in the graphs in Figs. 9D, E. No statistically significant differences in biochemical results were observed between the treated groups (GP2 and GP3) and the control group (GP1) (Figs. 9D, E).

**Fig. 10:**
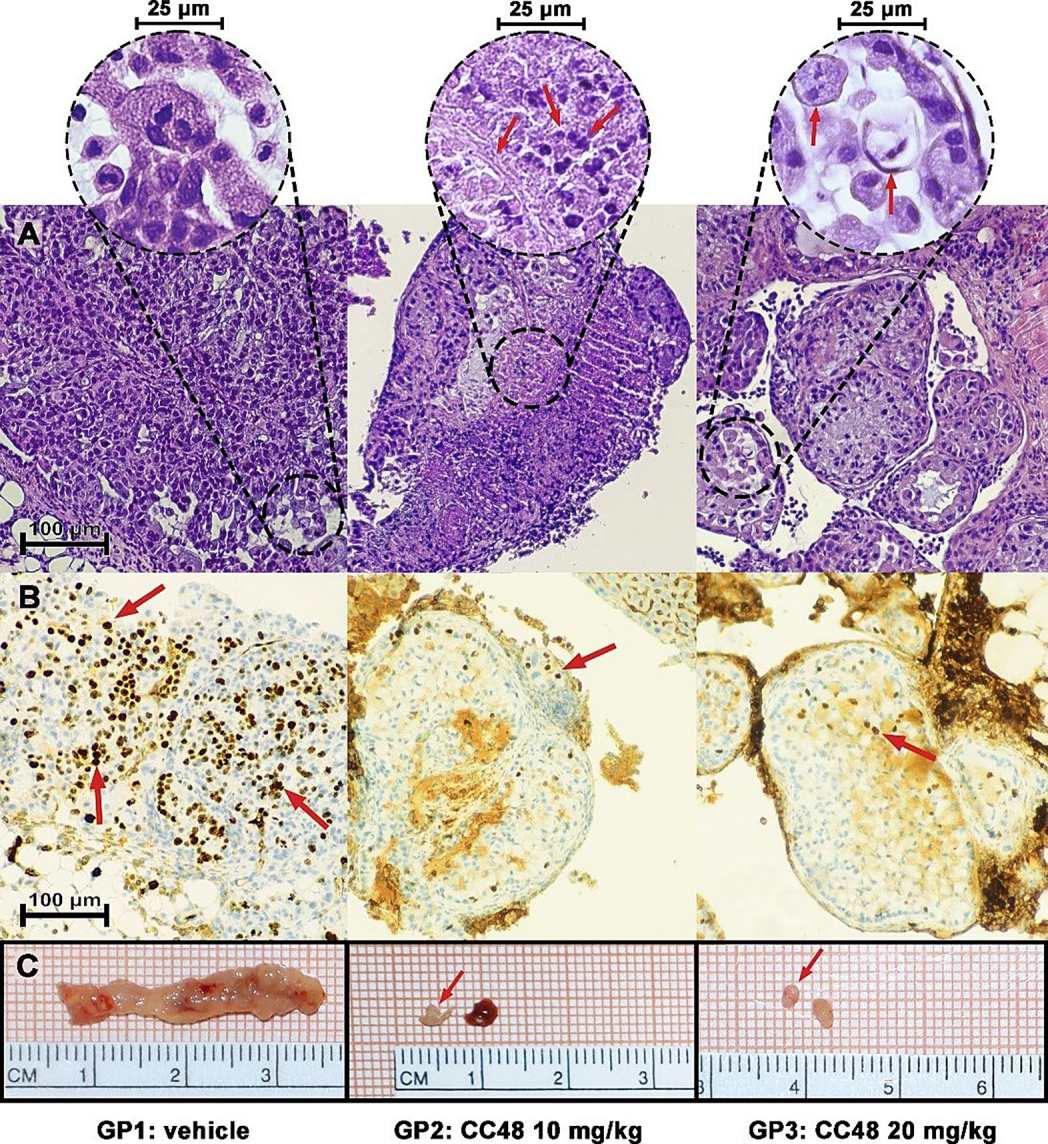
CC48 reduces tumor cells proliferation in treated mice **(A)** Histological sections stained with hematoxylin & eosin, representative of the tumor masses from each of the three experimental groups, vehicle-treated control group (GP1), group treated with 10 mg/Kg **CC48** (GP2) and group treated with 20 mg/Kg **CC48** (GP3) Lens: 10X. Bar: 100 μm. Circled areas enclose details of each histologic section taken at 40X magnification. In GP1 there are viable neoplastic cells, in GP2 the red arrow on the left indicates the necrotic area, and the two red arrows on the right indicate the lymphocytic infiltrate. In the GP3 group of mice, where the malignancy infiltrates the striated muscle of the murine diaphragm, the left arrow indicates a foamy histiocyte with fragmented nucleus, the right arrow indicates another foamy histiocyte with pyknotic nucleus (hydropic degeneration of tumor tissue) Lens: 40X. Bar: 25 μm. **(B)** At Immunohistochemical analysis of the intra-tumoral Ki67 marker as an index of cell proliferation, we calculated a mean of 35% of Ki67 stained nuclei in GP1 and a mean of 15–20% in GP2/GP3 (*p < 0.05 GP3 vs. GP1). Red arrows indicate some Ki67 positive nuclei. Lens: 10X. Bar: 100 μm. **(C)** The sampled tumor masses were photographed on graph paper. Images are representative of each treatment group

Hematoxylin and eosin (H&E) staining confirmed the development of tumors from the N87 tumor line cells in the mouse peritoneal cavity. H&E staining revealed tumors adhering to various sites including the stomach, liver, spleen or pancreas, all secondary sites of GC disease [30]. In some cases, infiltration of tumor cells into the same organs was observed, in others the tumor mass remained detached in the peritoneal cavity. Panels A of Fig. 10 showed the staining of tissue sections from three tumor explants, i.e. from the control group (GP1), the group treated with the CB2R agonist **CC48** at a concentration of 10 mg/Kg (GP1) and the group treated with the same compound at 20 mg/Kg, twice the previous concentration (GP2). Tumor cells from vehicle-treated mice show active proliferation, as evidenced by the high percentage (35%) calculated as the modal value of nuclei stained with the antibody that recognizes Ki67, a well-known proliferation marker commonly used by pathologists to express the degree of tumor proliferation (Fig. 10B). By contrast, tumor cells from **CC48**-treated mice at both concentrations show extensive areas of necrosis, with inflammatory and apoptotic elements. All these elements are clearly visible on analysis of the sections at higher magnification (40X), which in the sections from **CC48**-treated mice revealed numerous necrotic areas with phlogistic infiltrate, pyknotic nuclei approaching expulsion leading to fibrotic replacement. Furthermore, hydropic degeneration of tumor tissue with foamy histiocytes can also be seen in **CC48**-treated tumor tissues. Interestingly, in the tumor tissue staining of **CC48**-treated mice, a fibrotic replacement process was triggered, with the presence of pyknotic nuclei, as seen in the H&E staining of tumor tissue from the GP3 group of mice, where the malignancy infiltrates the striated muscle of the murine diaphragm. Both **CC48** concentrations also reduce the percentage of Ki67-stained nuclei to 15–20% (*p < 0.05 GP3 vs. GP1) (Fig. 10B). The histological sections shown in panels A and B were taken from the explanted masses at sacrifice and photographed on graph paper as shown in panels C of Fig. 10.

### CC48 reduces circulating levels of inflammatory factors

**Fig. 11:**
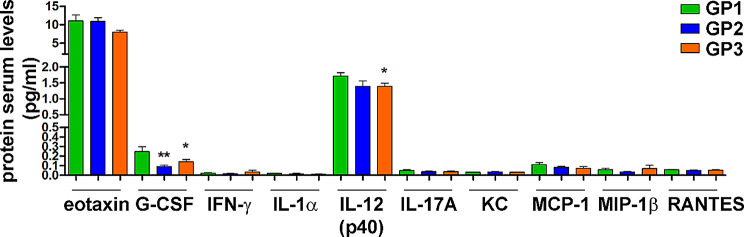
CB2R agonist CC48 reduces circulating levels of specific inflammatory cytokine. Serum levels of eotaxin, G-CSF, IFN-γ, IL-1α, IL-12 (p70), IL-17 A, GRO/KC (CXCL1), MCP-1 (CCL2), MIP-1β and RANTES were measured in serum samples and compared within the three experimental groups (GP1: vehicle; GP2: 10 mg/Kg CC48; GP3: 20 mg/Kg CC48). For each analyte, the value is the average of the six mice in each group ± SD ed expressed as pg/mL.*p < 0.05; **p < 0.01

The inflammatory and immune response was also investigated by assessing the levels of a panel of circulating markers related to the murine inflammatory and immune response in nude mice, lacking the thymus and with depleted mature T lymphocytes. A panel of 23 cytokines, chemokines and growth factors, mediating inflammation and immunity, was selected. Changes in the circulating levels of specific markers in the three experimental groups were correlated with the response to treatment with the **CC48** ligand. Of the 23 molecules analysed by the bio-plex immunoassay, 10 were expressed at detectable levels in the sera of each of the six mice in each of the three groups. As shown in Fig. 11, the levels of the chemokine eotaxin tended to decrease after treatment with the highest dose of **CC48** (GP3) compared to those in the mice in the control group (GP1), while a significant decrease was already observed at the lowest dose of the compound (GP2, **p < 0.01) and was maintained at the highest dose (GP3, *p < 0.05) for the growth factor G-Colony Stimulating Factor (G-CSF). Finally, for the regulatory isoform p40 of InterLeukin-12 (p40IL-12), a significant decrease (*p < 0.05) was observed in the sera of mice in the GP3 group compared to those in the GP1 group. For all other markers detected, there was no change in expression levels in the three experimental groups.

## Discussion

Gastric Cancer is often diagnosed at an advanced or metastatic stage, when surgery is no longer an option [1, 2]. Treatments are limited to combining two or more chemotherapy (CHT) agents, which are highly toxic and induce resistance mechanisms [3]. A better understanding of GC molecular hallmarks has increased the number of targeted agents, allowing the identification of personalized and tailored therapies [3–5]. Although these drugs have reduced the toxic effects of standard CHT, they have not fully addressed many of the challenges associated with resistance, and new efforts are needed to identify new targetable molecules and biomarkers predictive of therapy outcome.

In this context, CB2R is emerging as a pivotal biomarker, as it is overexpressed in inflammatory stages that are often associated with the early onset of cancer [9–14]. This study aimed to propose a multitarget therapeutic approach that combines both direct and indirect activation of CB2R as a novel strategy to counteract the onset and progression of GC. To this end, we evaluated the antitumor activity of three CB2R agonists from our in-house library, characterized by either dual-target activity (CB2R agonism and FAAH inhibition) or single-target CB2R agonism, each with a distinct pharmacodynamic profile. Additionally, two reference compounds were included: the CB2R agonist **1** and the CB2R antagonist **AM630** [16–20].

Among the compounds tested, the dual CB2R/FAAH ligands **CC48** and **Fi9** were selected for their high affinity and good selectivity for CB2R over CB1R. Meanwhile, **ASF151** was initially characterized as a single-target CB2R agonist due to its strong CB2R affinity and complete lack of CB1R binding [21, 22]. However, given the structural similarity among these compounds and their shared ability to modulate the balance between pro- and anti-inflammatory cytokine production, we further evaluated the FAAH inhibitory profile of **ASF151**. Our findings revealed that **ASF151** also exhibits FAAH inhibitory activity comparable to the multitarget ligands **CC48** and **Fi9**, suggesting a previously unrecognized dual mechanism of action. In contrast, no significant FAAH inhibition was observed for the two reference compounds, CB2R agonist **1** and the antagonist **AM630**. Among the three ligands, **CC48** demonstrated the highest CB2R affinity and functional activity. In some of the experiments presented, **AM630** showed effects similar to those induced by the other CB2R agonists tested. This supports earlier studies suggesting that **AM630**, a CB2R antagonist/inverse agonist, can exhibit CB2R agonist-like behaviour under certain conditions, likely due to off-target interactions [20, 31, 32]. The dualism highlights the complexity of CB2R-mediated mechanisms and is in line with recent findings that cannabinoid receptor ligands may elicit effects that do not always fit a strict agonist-antagonist dichotomy. Further studies are needed to fully elucidate these interactions as cannabinoid research evolves.

To evaluate the multi-target strategy as a novel approach to inhibiting GC tumour progression, we determined the cytotoxicity of each of the three dual drugs, together with reference compounds, in four GC cell lines characterised by different CB2R expression levels. The percentage of vitality and the relative IC_50_ values differed between the ligands in the same cell line, nonetheless there was no significant correlation with CB2R expression. Each compound’s inhibitory action was investigated at 1 µM and 10 µM, sub-cytotoxic concentrations that highlighted the activity of the CB2R ligands in the studied cellular processes.

One of these models was resistant to the cytotoxic action of the chemotherapeutic agent PTX, a taxane known as antimitotic drug that blocks the disassembly of interphase microtubules, leading to cell cycle arrest [33–35]. Although taxanes are a widely used and effective ‘weapon’ in cancer therapy, resistance to these drugs is observed in a large percentage of cases [34]. Taxane-mediated resistance is a process involving multiple intracellular signalling cascades and is still not fully understood. A previous study, investigating the hallmarks of PTX-mediated resistance through a comparative analysis of two taxane-resistant cell lines and their sensitive counterparts, demonstrated that the P-gp efflux pump, a key MDR transporter, plays a central role in the resistance mechanism [27]. The present study also aimed to investigate the effects of these compounds on the molecular processes contributing to PTX-mediated resistance in GC lines. The PTX-resistant HGC27 line was a useful tool to study some of the mechanisms involved in resistance induced by continuous treatment with low chemotherapeutic agent concentrations [27]. Among the three dual drugs selected from our CB2R ligand library, **CC48** stood out as the only one capable of interacting with P-gp with an EC_50_ of 7.75 µM. This interaction could be beneficial in reversing P-gp-mediated MDR.The increase in anti-tumor activity of **CC48** in combination with PTX found in several assays, mainly in CHT-resistant cells, may be partly related to the ability of **CC48** to overcome MDR by interacting with P-gp. Similarly, a synergy was observed between PTX and the two reference compounds **AM630** and **compound 1**, which also have an interaction profile with P-gp (EC_50_ = 16.8 µM and 24.7 µM for **AM630** and **compound 1**, respectively).

Noteworthily CB2R ligand treatments significantly impacted the expression and/or activation of key proteins in the PI3K/Akt/mTOR and ERK1/2 pathways in all cell lines tested, suggesting their involvement in the inhibitory action of CB2R ligands on cell growth [35].Compound **CC48**, compared to the dual agonists **Fi9** and **ASF151**, as well as the reference **compounds 1** and **AM630**, proved particularly effective in reducing the expression levels of almost all the analysed molecules, as well as reducing their activation by modulating the phosphorylation status of some of their specific target sites.

The percentage of actively proliferating cells (Ki67+) did not decrease significantly following treatment with CB2R ligands, and there were no particular differences between single- and dual-target compounds. However, a potentiation of inhibition due to PTX was observed following combination treatments. Notably, inhibition was observed following the combination treatment of PTX and compounds **1** and **CC48** in PTX-resistant cells.

To further investigate the anti-tumor profile of CB2R ligands, other mechanisms such as ROS production and autophagy were also explored. Measuring ROS in tumor cells may help assess the level of oxidative stress, which is often elevated in cancer and can contribute to tumor progression. Therefore, after 48 h of treatment with all CB2R ligands, only **CC48** of the CB2R agonists induced ROS production in HCG27-sensitive cells and **ASF151** in the HCG27-resistant counterpart. As previously reported, ROS induction was also observed in both HGC27 cell lines following **AM630** treatment [20, 32–34].

Cannabinoids have been reported to activate autophagy by inducing endoplasmic reticulum stress and inhibiting the Akt/mTORC1 complex in glioma [36].Increased autophagy induces apoptosis in solid cancer cells [37–39], pointing the way to the exploitation of CBR ligands as effective inducers of cancer cell death. However, it is still poorly understood whether the effect is mediated by CB1R or CB2R activators or inhibitors [41]. In our hands, the CB2R dual drugs **CC48** and **Fi9**, which were also the best CB2R agonists in terms of affinity and activity, were the most potent inducers of autophagy, while **ASF151** (less potent in terms of CB2R affinity and activity) was the least potent inducer of autophagy among our ligands. The pure CB2R reference agonist **1** had no effects on autophagic proteins, while the CB2R antagonist **AM360** induced autophagy. By crossing these data, it seems that the activation of CB2R together with FAAH inhibition are the conditions associated with the strongest induction of autophagy.

It is well known that the induction of autophagy can be a trigger for apoptotic cell death [41]. The regulation of the apoptotic process by CB2R ligands has been extensively studied both by cytofluorometry, revealing the percentage of apoptotic cells by the binding of Annexin V protein to phosphatidylserine exposed on the apoptotic cell membrane, and by the expression of major apoptotic proteins involved in the pro-apoptotic cascade JNK/JUN and the activation of the two cleavage active forms of caspase 3 and 7 [40].Both of these analyses detected a significant induction of the apoptotic process in PTX-sensitive cells, even with a single treatment with the CB2R ligand. The reference compounds and **CC48** potentiated the effect of PTX, as shown by analysis of caspase 3/7 activation status. All the compounds were effective in inducing the apoptotic process, even in PTX-resistant cells, and there was an outstanding synergy of PTX with CB2R reference ligands and **CC48** in combination treatments.

**Fig. 12:**
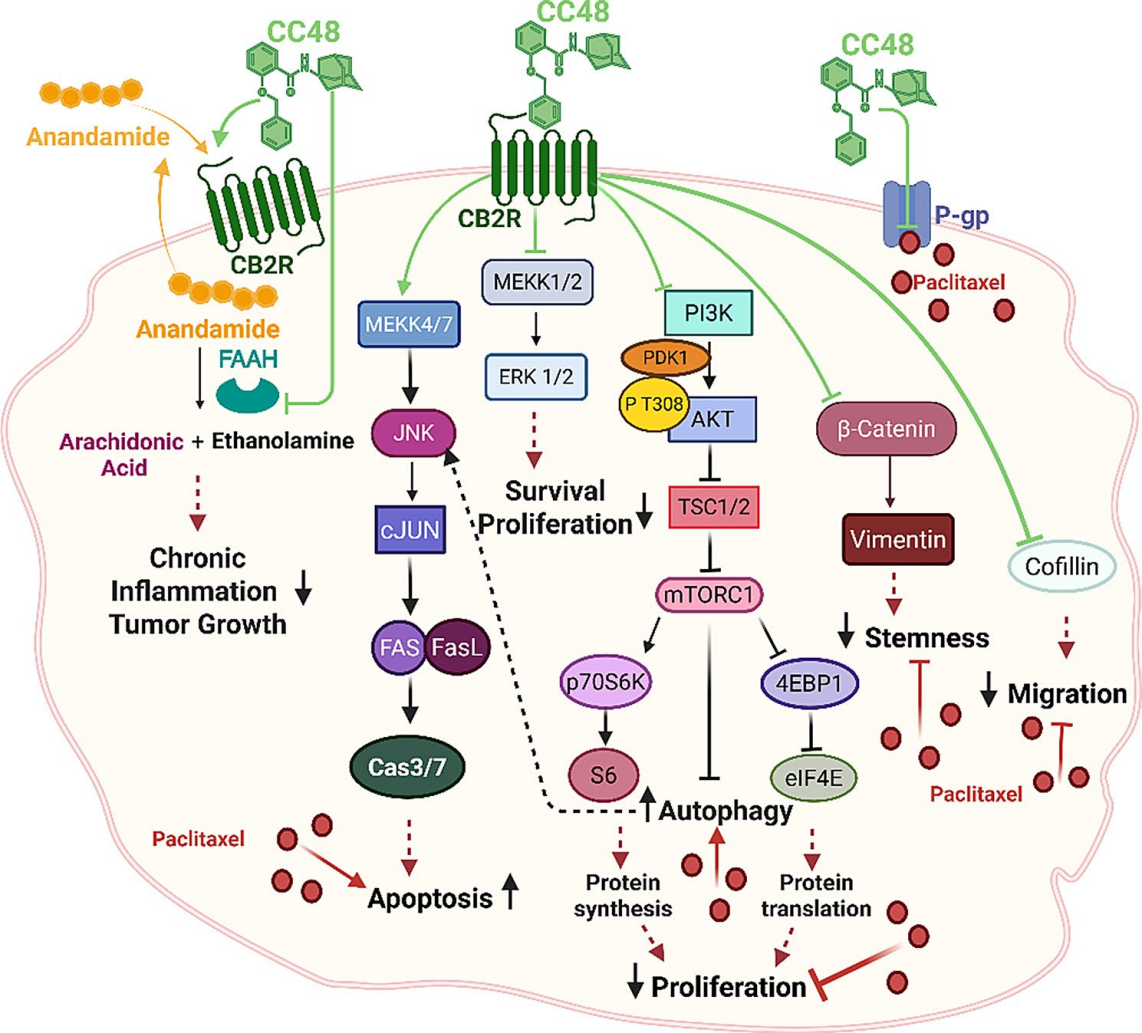
Effects of CC48 on tumour growth, selected as a first-in-class cannabinoid receptor type 2 (CB2R) multi-target agent. The agonist **CC48** exerts its antitumour activity in gastric cancer cell models by (i) binding to CB2R; (ii) inhibiting the FAAH enzyme; (iii) interacting with the P-gp protein responsible for the efflux of the chemotherapeutic drug PTX from the tumor cell. The main molecular pathways regulated by **CC48** and involved in proliferation, autophagy and apoptosis were depicted. Previous studies have shown that the same pathways are involved in the anti-tumor activity of PTX. **CC48** enhances PTX-induced cell growth inhibition, even in chemotherapy-resistant cell models. Image created in https://BioRender.com

The reference compounds and **CC48** were also particularly effective in reducing the migration rate of both PTX-sensitive and PTX-resistant GC lines. This was supported by the significant inhibition of cofillin, a protein involved in cytoskeletal remodelling [41]. The action of **CC48** was particularly effective compared to the other compounds tested in reducing the activation of β-catenin, resulting in decreased expression levels of vimentin, a protein involved in the epithelial-mesenchymal transition [42]. Characterization of the PTX-resistant HGC27 line revealed that overexpression of factors such as VEGFA, VEGFC and Angiopoietin 2 represented one of the key hallmarks of drug resistance [27]. The action of these growth factors is not only involved in tumor angiogenesis, but is also crucial in supporting tumor cell growth through an autocrine mechanism [43]. Therefore, the effects of the CB2R ligands studied on the VEGFA secretion process were investigated. The results obtained in both PTX-sensitive and PTX-resistant cell lines clearly showed that only treatments with **CC48** and **compound 1** resulted in a significant reduction of VEGF secretion in the culture medium. In Fig. 12 are summarized the main molecular pathways regulated by the first-in-class agonist, **CC48**, involved in proliferation, migration and apoptosis.

Compound **CC48** showed best promise in an in vivo GC model, following evaluation of all in vitro findings. The efficacy of the **CC48** compound was evaluated in orthotopic mouse models obtained by intraperitoneal inoculation of NCI-N87_LUC cells [29]. Analysis of tumour masses revealed significant tumour volume reduction in mice treated with **CC48** at both concentrations, with a further reduction in tumour mass size in mice treated with the highest dose of the compound (GP3, 20 mg/kg). No differences in body weight were observed between the control group and the treated groups. Similarly, no differences were seen in kidney biochemistry and urinary parameters.

H&E staining confirmed the development of tumors from N87 GC cellsin the mouse peritoneum. Tumors adhered to various sites including the stomach, liver, spleen or pancreas, all secondary sites of GC disease [30]. In some cases, there was infiltration of tumor cells into the same organs; in others, the tumor mass remained avulsed in the cavity. Histological analysis of the explanted tumor masses revealed the characteristics of gastric adenocarcinoma with liver metastases, from which the N87-NCl cells were derived. Ki67 staining revealed a high percentage of nuclei positive for the actively proliferating cell marker in tumors derived from control mice. By contrast, **CC48**-treated mouse tumor cells show extensive areas of necrosis with inflammatory and apoptotic elements, clearly visible on high-magnification analysis. The sections reveal numerous necrotic areas with inflammatory cells and pyknotic nuclei, leading to fibrotic replacement [44]. Furthermore, hydropic degeneration of tumor tissue can also be seen. These regression elements were consistent with a reduction in the percentage of Ki67-positive nuclei to 15–20% observed with both concentrations of **CC48**.

Inflammatory and immune responses were investigated by assessing levels of a panel of circulating markers related to murine inflammation and immunity. A panel of 23 inflammation and immunity markers was analysed. The changes in the levels of these markers were correlated with their response to treatment with the **CC48** ligand. Treatment with the highest dose of **CC48** (GP3) led to a non-significant decrease in the levels of the chemokine eotaxin compared to the control group (GP1). G-CSF showed a significant decrease at the lowest dose (GP2, **p < 0.01) and was also maintained at the highest dose (GP3, *p < 0.05). A significant decrease in sera for the subunit p40 IL-12 (*p < 0.05) from the GP3 group was also observed as compared to the GP1 group. No other change in marker expression was seen. Eotaxins are small proteins that act on eosinophils and are involved in carcinogenesis. This chemokine may be a good cancer biomarker for diagnosing, predicting treatment response and monitoring inflammation [45–50].The growth factor G-CSF promotes the production, maturation, and migration of neutrophils from the bone marrow to the bloodstream [51]. The interleukin IL-12(p40) acts as a macrophage chemoattractant together with IL-12(p70) to induce the initial immune responses [52]. Therefore, the results were consistent with the hypothesis that tumor development in the nude mouse leads to an increase in specific cytokines/chemokines responsible for an early and rapid immune response by immune cells of the myeloid lineage. The reduction in tumor mass in **CC48**-treated mice would explain the reduction in blood levels of these cytokines. This finding was related to the presence of cells belonging to a later phase of the immune response, mediated by plasma cells and histiocytes, in the tumor tissue of **CC48**-treated mice, while only a few eosinophils and basophils were identified in the tumors of the control mice.

## Conclusion

In conclusion, the activity exhibited by **CC48** across all investigated pathways supports a multitarget approach. This includes both direct and indirect activation of CB2R, via CB2R agonism and FAAH inhibition, along with interaction with the multidrug resistance protein P-gp. Together, these mechanisms represent a promising strategy to address gastric cancer, particularly in its drug-resistant forms. Furthermore, **CC48** was well-tolerated and could offer a therapeutic window for extended treatment or maintenance therapy. The anti-tumor activity studied in in vitro and in orthotopic models of human GC is complemented by the ability to overcome PTX resistance mechanisms, as supported by data obtained in a cellular model of chemotherapy resistance. The dual valency of this compound, combined with its low toxicity, has led to a patent application (*Italian Patent Application IT102024000029760*,* 23/12/2024*). The counteraction of the PTX-mediated resistance by **CC48** in vitro confirms the synergy between the two compounds, providing the basis for combined treatment in animal models.

## Electronic supplementary material

Below is the link to the electronic supplementary material.

Supplementary Material 1: Expression of CB1R and CB2R in AGS, KATOIII, HGC27-S/R and NCl-N87. **A**) The levels of mRNA were expressed as DDCq in the different cell lines. mRNA expression was normalized to the housekeeping gene GAPDH. Data were mean ± SD (n=3). **B**) Immunoblotting of the two CB receptors in GC cell lines. Whole cell lysates were subjected to immunoblotting for the indicated proteins. Actin was used as an equal loading control. The figure was representative of 3 independent experiments and the graphs displayed below reported the relative expression of the proteins compared to expression in AGS cells. Data were mean ± SD (n=3).

Supplementary Material 2: Differences in IC50 values among CB2R ligands within each cell line, and for a given compound across different cell lines at 48 hours. **A**) Statistically significant differences between IC_50_ of all CB2R compounds in each GC cell line: AGS, HGC27-S, HGC27-R and NCI-N87 calculated with one-way ANOVA analysis. **B**) Statistically significant differences in IC_50_ values for the same CB2R compound (AM630, CC48, Fi9, ASF151 and compound 1) across different cell lines assessed with the two-way ANOVA. *p < 0.05; **p < 0.01; ***p < 0.001; ****p<0.0001.

Supplementary Material 3: Western blot analysis of expression and activation levels of key proteins involved in cell proliferation. Representative western blotting analyses performed in HGC27-S/R and AGS cells of the expression of the phosphorylated and total forms of TSC2, PI3K, P70, Akt, S6, 4EBP1 and ERK1/2 after 48 hours of treatment with the two different concentrations of the compounds **AM630**, **CC48**, **Fi9**, **ASF151** and **compound 1**. The expression levels of each of the investigated proteins were normalized to the actin level.

Supplementary Material 4

## Data Availability

The relevant datasets generated and/or analysed during the current study are available in the [FIGSHARE] repository, https://doi.org/10.6084/m9.figshare.28740560.v1. All the other datasets used and/or analysed during the current study are available from the corresponding author on reasonable request.

## Abbreviations

GC: Gastric Cancer
PTX: Paclitaxel
GPCR: G protein-coupled receptors
CRC: colorectal cancer
CBD: Cannabidiol
MTDLs: MultiTarget Directed Ligands
FAAH: Fatty Acid Amide Hydrolase
CB2R: cannabinoid receptor subtypes 2
CB1R: cannabinoid receptor subtypes 1
P-gp: glycoprotein-P
ROS: Reactive Oxygen Species
DCF-DA: 2’,7’-dichlorofluorescein diacetate
HGC27-S or HGC27-R: PTX-Sensible or -Resistant HGC27
Ki: Inhibitor costant
SEM: The Standard Error of the Mean
EC50: half maximal (50) Effective Concentration
IC50: Half Maximal Inhibitory Concentration
DMEM: Dulbecco’s Modified Eagle Medium
RPMI: Roswell Park Memorial Institute
NCl-N87_LUC: NCl-N87_luciferase
H&E: Haematoxylin and Eosin
IHC: ImmunoHistoChemistry
BW: Body Weight
AST: Aspartate Transferase
ALT: Alanine Aminotransferase
Cre: Creatinine
GFR: Glomerular Filtration Rate
G-CSF: G-Colony Stimulating Factor
p40IL-12: isoform p40 of InterLeukin-12
