# Supplementary figures and images for: CC48 a new CB2R agonist/FAAH inhibitor dual drug blocks gastric cancer progression and overcomes paclitaxel resistance
Source: J Exp Clin Cancer Res. 2025 Jul 16;44:209. doi: 10.1186/s13046-025-03476-7 (PMC12265377; doi:10.1186/s13046-025-03476-7)

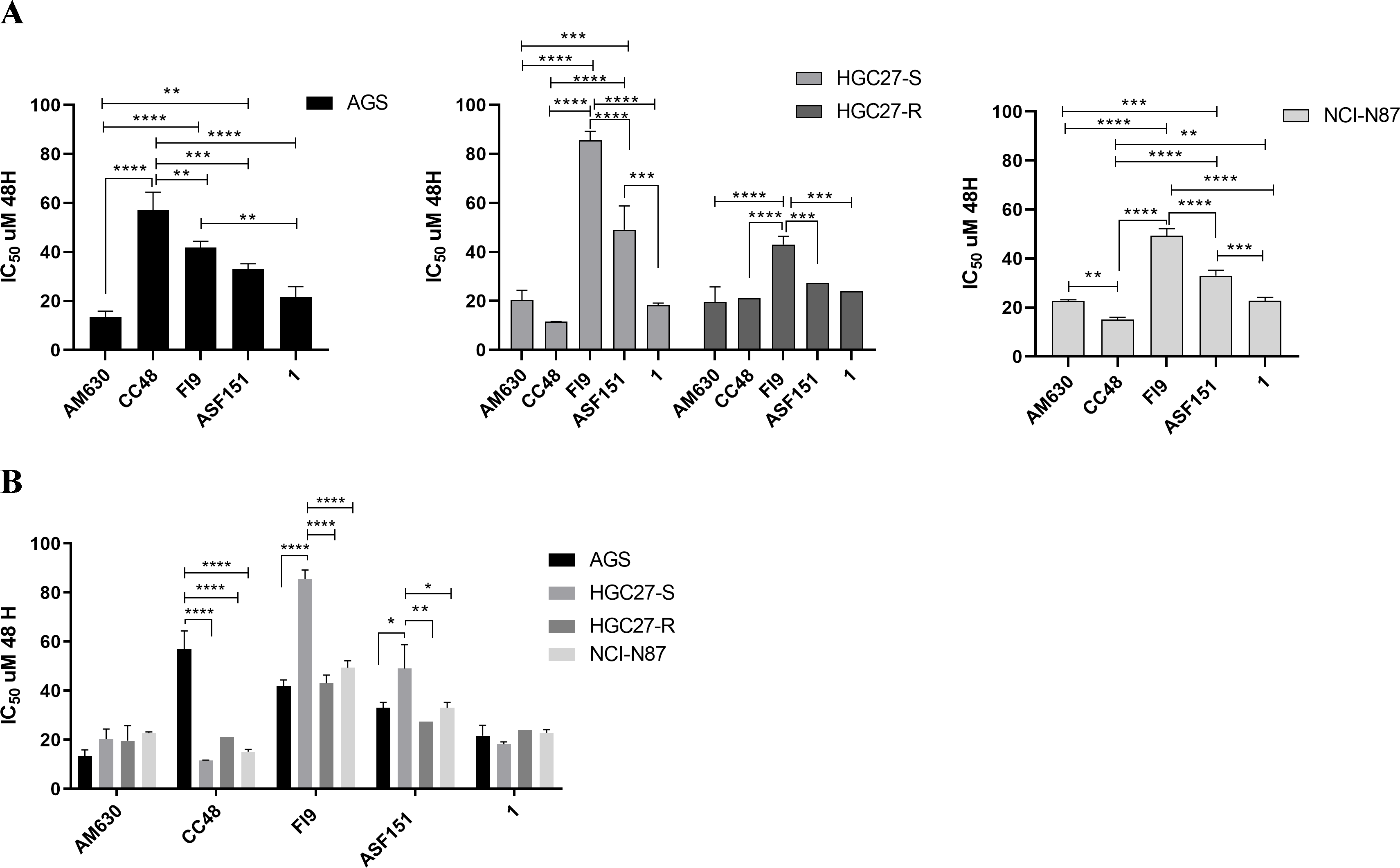

Supplement: Supplementary file 1 — Supplementary Material 1: Expression of CB1R and CB2R in AGS, KATOIII, HGC27-S/R and NCl-N87. A) The levels of mRNA were expressed as DDCq in the different cell lines. mRNA expression was normalized to the housekeeping gene GAPDH. Data were mean ± SD (n=3). B) Immunoblotting of the two CB receptors in GC cell lines. Whole cell lysates were subjected to immunoblotting for the indicated proteins. Actin was used as an equal loading control. The figure was representative of 3 independent experiments and the graphs displayed below reported the relative expression of the proteins compared to expression in AGS cells. Data were mean ± SD (n=3). [file 13046_2025_3476_MOESM1_ESM.png]

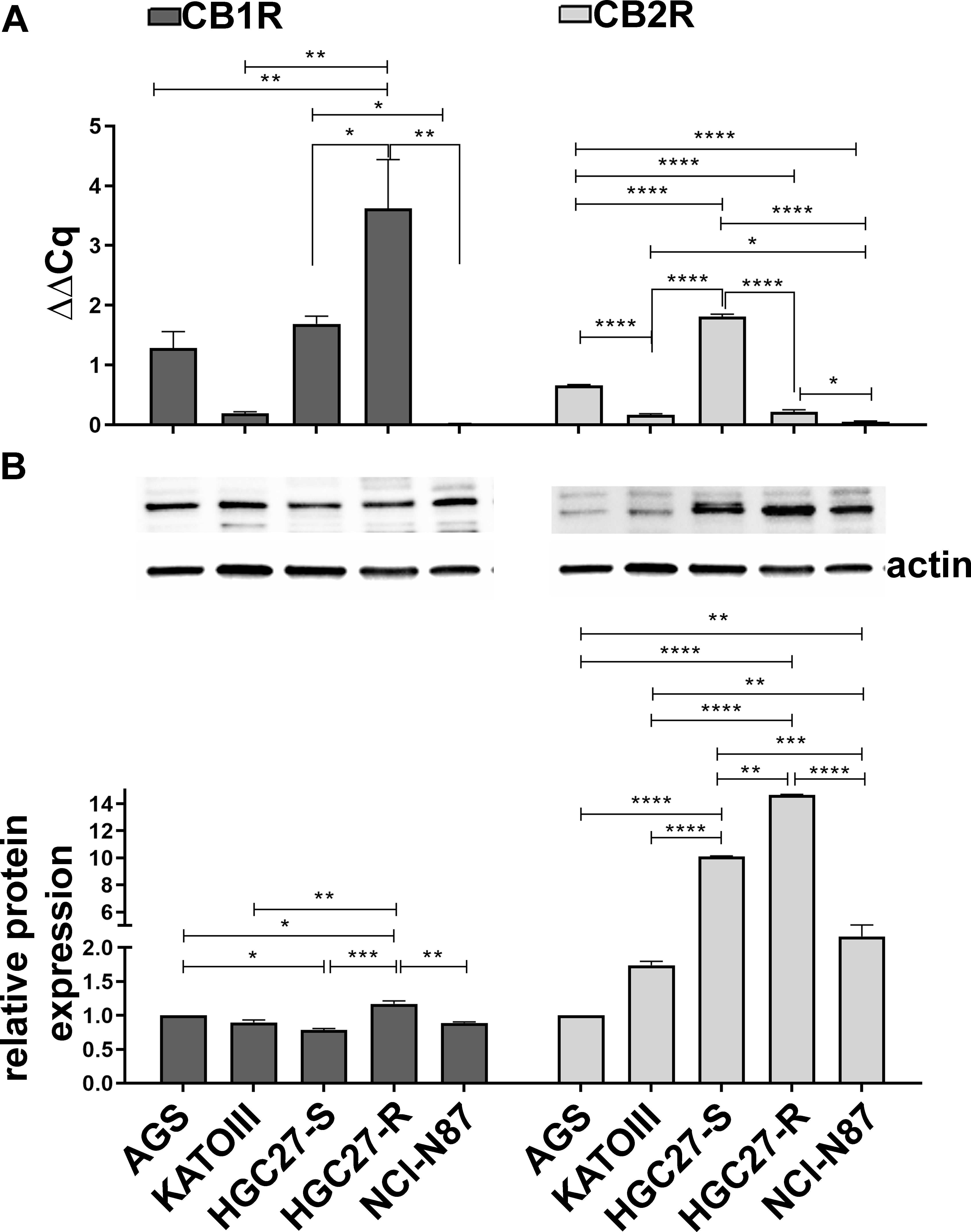

Supplement: Supplementary file 2 — Supplementary Material 2: Differences in IC50 values among CB2R ligands within each cell line, and for a given compound across different cell lines at 48 hours. A) Statistically significant differences between IC50 of all CB2R compounds in each GC cell line: AGS, HGC27-S, HGC27-R and NCI-N87 calculated with one-way ANOVA analysis. B) Statistically significant differences in IC50 values for the same CB2R compound (AM630, CC48, Fi9, ASF151 and compound 1) across different cell lines assessed with the two-way ANOVA. *p < 0.05; **p < 0.01; ***p < 0.001; ****p<0.0001. [file 13046_2025_3476_MOESM2_ESM.png]

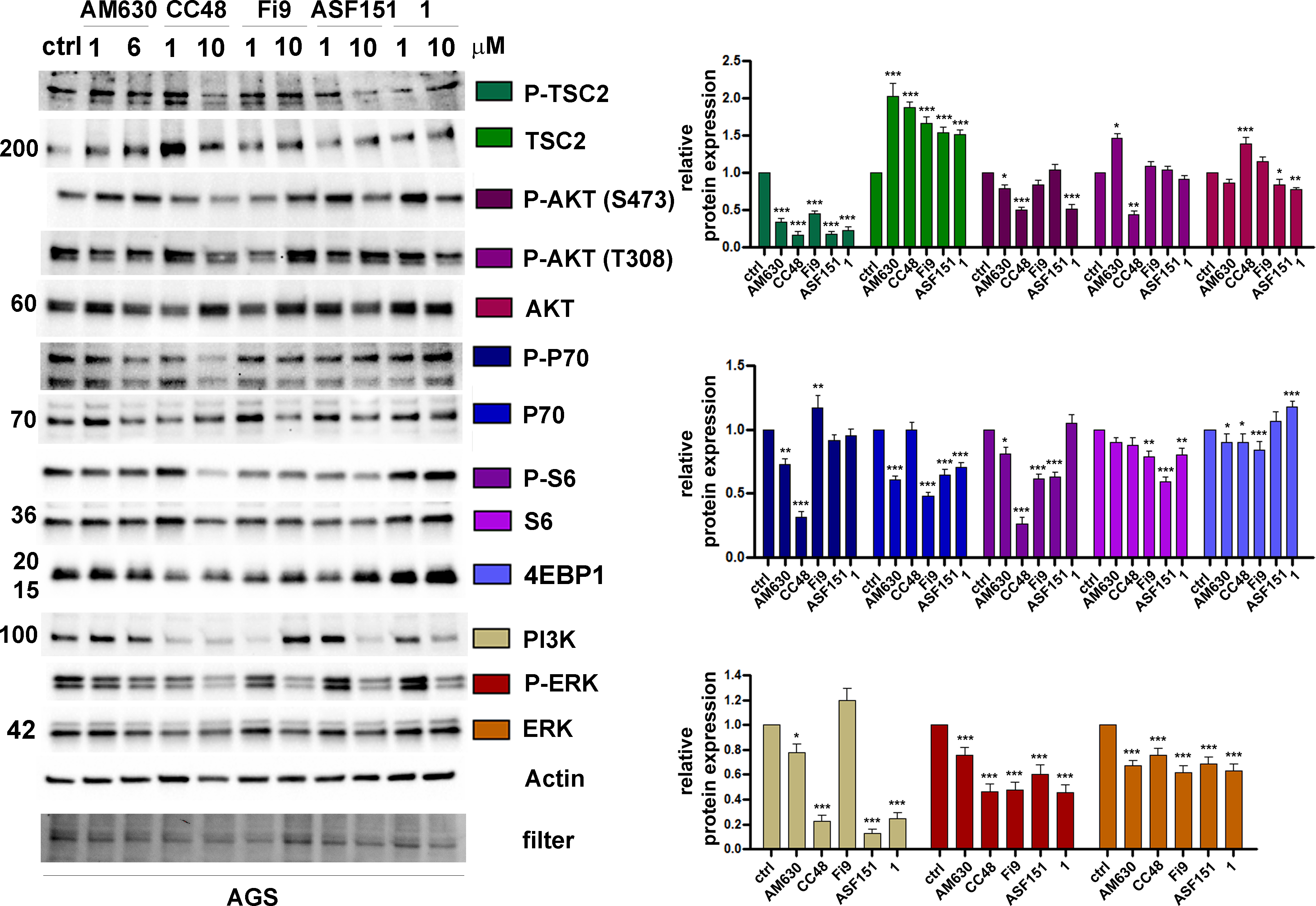

Supplement: Supplementary file 3 — Supplementary Material 3: Western blot analysis of expression and activation levels of key proteins involved in cell proliferation. Representative western blotting analyses performed in HGC27-S/R and AGS cells of the expression of the phosphorylated and total forms of TSC2, PI3K, P70, Akt, S6, 4EBP1 and ERK1/2 after 48 hours of treatment with the two different concentrations of the compounds AM630, CC48, Fi9, ASF151 and compound 1. The expression levels of each of the investigated proteins were normalized to the actin level. [file 13046_2025_3476_MOESM3_ESM.png]
