# Supplementary material for: CC48 a new CB2R agonist/FAAH inhibitor dual drug blocks gastric cancer progression and overcomes paclitaxel resistance
Source: J Exp Clin Cancer Res. 2025 Jul 16;44:209. doi: 10.1186/s13046-025-03476-7 (PMC12265377; doi:10.1186/s13046-025-03476-7)
Supplement: Supplementary file 4 — Supplementary Material 4 [file 13046_2025_3476_MOESM4_ESM.docx]

**Table S1.** Cytotoxic activity of the CB2R ligands.

| **Cytotoxic activity**  IC_50_±SEM, μM^a^ | | |
| --- | --- | --- |
| **AGS cells** | | |
| ***compound*** | ***48h*** | ***72h*** |
| **AM630** | 13.4 ± 2.5 | 7.5 ± 1.2 |
| **CC48** | 57.0 ± 7.4 | 9.8 ± 1.5 |
| **Fi9** | 41.9 ± 2.5 | 7.5 ± 1.5 |
| **ASF151** | 33.0 ± 2.2 | 42.3 ± 2.2 |
| **1** | 21.6 ± 4.3 | 13.2 ± 1.2 |
| **HGC27-S cells** | | |
| **AM630** | 20.4 ± 4.0 | 13.69 ± 1.4 |
| **CC48** | 11.6 ± 0.10 | 14.52 ± 0.34 |
| **Fi9** | 85.5 ± 3.7 | 19.60 ± 0.83 |
| **ASF151** | 49.0 ± 9.8 | 30.07 ± 0.95 |
| **1** | 18.2 ± 0.96 | 18.78 ± 0.48 |
| **HGC27-R cells** | | |
| **AM630** | 19.61 ± 6.15 | 19.13 ± 0.93 |
| **CC48** | 21.01 ± 0.002 | 21.17 ± 0.41 |
| **Fi9** | 43.0 ± 3.40 | 39.0 ± 2.95 |
| **ASF151** | 27.39 ± 0.01 | 28.04 ± 0.63 |
| **1** | 24.0 ± 0.00 | 21.49 ± 0.85 |
|  | **NCI-N87 cells** |  |
| **AM630** | 22.72 ± 0.52 | 15.56 ± 0.26 |
| **CC48** | 15.09 ± 0.93 | 9.42 ± 0.22 |
| **Fi9** | 49.36 ± 2.83 | 19.22 ± 0.03 |
| **ASF151** | 33.06 ± 2.11 | 29.32 ± 3.84 |
| **1** | 22.76 ± 1.34 | 19.19 ± 1.54 |

^a^ Values are the mean ± SEM of three independent experiments performed in triplicate; half maximal (50) Inhibitory Concentration (IC_50_); The Standard Error of the Mean (SEM)

**Table S2.** Individual and average BLI measurements with outlier calculated with Rout test (Q1%).

| **Group** | **Treatment** | **ID** | **DAY 5** | **Rout Test**  **(Q 1%) DAY 5** | **DAY 7** | **DAY 10** | **DAY 14** | **DAY 17** | **DAY 24** | **DAY 31** | **DAY 35** | **DAY 38** | **DAY 41** | **Rout Test (Q 1%) DAY 41** |
| --- | --- | --- | --- | --- | --- | --- | --- | --- | --- | --- | --- | --- | --- | --- |
|  |  | **1** | 5,42E+06 | **outlier** | 6,83E+06 | 6,69E+06 | 1,14E+07 | 1,35E+07 | 1,98E+07 | 2,59E+07 | 2,60E+07 | 2,85E+07 | 2,91E+07 | **outlier** |
| **GP1** | **Vehicle** | **2** | 3,37E+05 |  | 9,82E+05 | 6,59E+05 | 4,52E+05 | 5,27E+05 | 4,23E+05 | 8,53E+05 | 1,39E+06 | 1,60E+06 | 2,97E+06 |  |
|  |  | **3** | 1,40E+05 |  | 2,61E+05 | 2,32E+05 | 3,28E+05 | 3,25E+05 | 8,62E+05 | 2,05E+06 | 3,77E+06 | 3,92E+06 | 4,80E+06 |  |
|  |  | **4** | 3,55E+04 |  | 5,39E+04 | 1,57E+04 | 1,50E+04 | 1,02E+04 | 1,09E+04 | 1,10E+04 | 1,56E+04 | 1,43E+04 | 1,37E+04 |  |
|  |  | **5** | 3,48E+04 |  | 9,25E+04 | 4,47E+04 | 3,01E+05 | 1,54E+05 | 4,20E+04 | 2,29E+05 | 1,31E+05 | 2,66E+05 | 5,36E+05 |  |
|  |  | **6** | 3,11E+03 |  | 4,57E+03 | 4,78E+04 | 4,15E+03 | 4,22E+03 | 5,14E+03 | 6,93E+03 | 4,47E+03 | 5,43E+03 | 5,46E+03 |  |
|  |  | **MEAN** | **1,10E+05** |  | **2,79E+05** | **2,00E+05** | **2,20E+05** | **2,04E+05** | **2,69E+05** | **6,29E+05** | **1,06E+06** | **1,16E+06** | **1,66E+06** |  |
|  |  | **SD** | **1,37E+05** |  | **4,05E+05** | **2,71E+05** | **2,00E+05** | **2,23E+05** | **3,75E+05** | **8,64E+05** | **1,62E+06** | **1,68E+06** | **2,14E+06** |  |
|  |  | **n** | **5** |  | **5** | **5** | **5** | **5** | **5** | **5** | **5** | **5** | **5** |  |
|  |  | **7** | 1,32E+06 | **Border-line** | 1,55E+06 | 1,30E+06 | 7,07E+05 | 8,99E+05 | 1,09E+06 | 1,51E+06 | 2,93E+06 | 4,74E+06 | 3,93E+06 | **outlier** |
| **GP2** | **FI-5 (10mg/kg)** | **8** | 5,58E+05 |  | 5,41E+05 | 4,73E+05 | 8,53E+05 | 1,29E+06 | 1,51E+06 | 1,43E+06 | 1,06E+06 | 1,04E+05 | 8,93E+05 |  |
|  |  | **9** | 1,13E+05 |  | 7,10E+04 | 4,20E+04 | 3,80E+04 | 6,23E+04 | 6,35E+04 | 1,11E+05 | 1,41E+05 | 1,11E+05 | 1,54E+05 |  |
|  |  | **10** | 4,14E+04 |  | 2,79E+04 | 1,70E+04 | 1,58E+04 | 1,12E+04 | 1,11E+04 | 1,60E+04 | 1,56E+04 | 9,13E+03 | 9,95E+03 |  |
|  |  | **11** | 9,93E+03 |  | 3,30E+03 | 2,56E+03 | 2,68E+03 | 1,95E+03 | 2,24E+03 | 3,40E+03 | 3,01E+03 | 2,90E+03 | 3,19E+03 |  |
|  |  | **12** | 3,23E+03 |  | 2,39E+03 | 2,69E+03 | 3,22E+03 | 1,85E+03 | 2,96E+03 | 3,65E+03 | 3,50E+03 | 2,69E+03 | 2,28E+03 |  |
|  |  | **MEAN** | **1,45E+05** |  | **1,29E+05** | **1,07E+05** | **1,83E+05** | **2,73E+05** | **3,18E+05** | **3,12E+05** | **2,44E+05** | **4,60E+04** | **2,12E+05** |  |
|  |  | **SD** | **2,35E+05** |  | **2,32E+05** | **2,05E+05** | **3,75E+05** | **5,68E+05** | **6,67E+05** | **6,24E+05** | **4,58E+05** | **5,63E+04** | **3,86E+05** |  |
|  |  | **n** | **5** |  | **5** | **5** | **5** | **5** | **5** | **5** | **5** | **5** | **5** |  |
|  |  | **13** | 1,03E+06 | **Border-line** | 7,47E+05 | 3,56E+05 | 1,23E+06 | 1,63E+06 | 1,67E+06 | 1,22E+06 | 2,08E+06 | 2,42E+06 | 1,34E+06 | **outlier** |
| **GP3** | **FI-5 (20mg/kg)** | **14** | 8,12E+05 |  | 4,61E+05 | 3,68E+05 | 5,29E+05 | 7,10E+05 | 1,15E+06 | 1,26E+06 | 1,71E+06 | 9,14E+05 | 3,58E+04 |  |
|  |  | **15** | 1,11E+05 |  | 2,67E+04 | 3,53E+04 | 3,27E+04 | 2,61E+04 | 3,46E+04 | 2,79E+04 | 4,56E+04 | 3,50E+04 | 3,11E+04 |  |
|  |  | **16** | 7,63E+04 |  | 5,08E+04 | 3,80E+04 | 1,72E+04 | 1,16E+04 | 3,03E+04 | 1,85E+04 | 2,63E+04 | 4,29E+04 | 2,06E+04 |  |
|  |  | **17** | 9,82E+03 |  | 3,79E+03 | 4,35E+03 | 3,88E+03 | 3,86E+03 | 8,48E+03 | 3,82E+03 | 3,77E+03 | 4,55E+03 | 6,77E+03 |  |
|  |  | **18** | 4,50E+03 |  | 9,41E+03 | 4,07E+03 | 7,83E+03 | 4,05E+03 | 6,74E+03 | 5,79E+03 | 5,12E+03 | 6,33E+03 | 5,88E+03 |  |
|  |  | **MEAN** | **2,03E+05** |  | **1,10E+05** | **8,98E+04** | **1,18E+05** | **1,51E+05** | **2,46E+05** | **2,64E+05** | **3,58E+05** | **2,00E+05** | **2,00E+04** |  |
|  |  | **SD** | **3,44E+05** |  | **1,97E+05** | **1,56E+05** | **2,30E+05** | **3,13E+05** | **5,05E+05** | **5,58E+05** | **7,56E+05** | **3,99E+05** | **1,37E+04** |  |
|  |  | **n** | **5** |  | **5** | **5** | **5** | **5** | **5** | **5** | **5** | **5** | **5** |  |

Tumor growth was evaluated by IVIS spectrum PerkinElmer. Day 21, 28, 38 and 41; ID: 1, 7 and 13 have been excluded from the analysis.

**Table S3.** Tumor volume expressed as mean and standard deviation calculated in each experimental Group (GP1, GP2 and GP3).

Dunnett's multiple comparison statistical test was applied. **p≤0,01; ***p≤0,001.
